# The transcription factor SPL13 mediates strigolactone suppression of shoot branching by inhibiting cytokinin synthesis in *Solanum lycopersicum*

**DOI:** 10.1093/jxb/erad303

**Published:** 2023-07-28

**Authors:** Shangyu Chen, Xuewei Song, Qixiang Zheng, Yuqi Liu, Jingquan Yu, Yanhong Zhou, Xiaojian Xia

**Affiliations:** Department of Horticulture, Zijingang Campus, Zhejiang University, Hangzhou 310058, PR China; Department of Horticulture, Zijingang Campus, Zhejiang University, Hangzhou 310058, PR China; Department of Horticulture, Zijingang Campus, Zhejiang University, Hangzhou 310058, PR China; Department of Horticulture, Zijingang Campus, Zhejiang University, Hangzhou 310058, PR China; Department of Horticulture, Zijingang Campus, Zhejiang University, Hangzhou 310058, PR China; Hainan Institute, Zhejiang University, Sanya 572025, PR China; Key Laboratory of Horticultural Plants Growth, Development and Quality Improvement, Ministry of Agriculture and Rural Affairs of China, Hangzhou 310058, PR China; Department of Horticulture, Zijingang Campus, Zhejiang University, Hangzhou 310058, PR China; Hainan Institute, Zhejiang University, Sanya 572025, PR China; Department of Horticulture, Zijingang Campus, Zhejiang University, Hangzhou 310058, PR China; Hainan Institute, Zhejiang University, Sanya 572025, PR China; University of Auckland, New Zealand

**Keywords:** Cytokinin, plant architecture, shoot branch, SPL13, strigolactone

## Abstract

Plant architecture imposes a large impact on crop yield. *IDEAL PLANT ARCHITECTURE 1* (*IPA1*), which encodes a SQUAMOSA PROMOTER BINDING PROTEIN-LIKE (SPL) transcription factor, is a target of molecular design for improving grain yield. However, the roles of SPL transcription factors in regulating tomato (*Solanum lycopersicum*) plant architecture are unclear. Here, we show that the expression of *SPL13* is down-regulated in the lateral buds of strigolactone (SL)-deficient *ccd* mutants and is induced by GR24 (a synthetic analog of SL). Knockout of *SPL13* by CRISPR/Cas9 resulted in higher levels of cytokinins (CKs) and transcripts of the CK synthesis gene *ISOPENTENYL TRANSFERASES 1* (*IPT1*) in the stem nodes, and more growth of lateral buds. GR24 suppresses CK synthesis and lateral bud growth in *ccd* mutants, but is not effective in *spl13* mutants. On the other hand, silencing of the *IPT1* gene inhibited bud growth of *spl13* mutants. Interestingly, SL levels in root extracts and exudates are significantly increased in *spl13* mutants. Molecular studies indicated that SPL13 directly represses the transcription of *IPT1* and the SL synthesis genes *CAROTENOID CLEAVAGE DIOXYGENASE 7* (*CCD7*) and *MORE AXILLARY GROWTH 1* (*MAX1*). The results demonstrate that SPL13 acts downstream of SL to suppress lateral bud growth by inhibiting CK synthesis in tomato. Tuning the expression of *SPL13* is a potential approach for decreasing the number of lateral shoots in tomato.

## Teaser

SPL13 acts downstream of strigolactone to suppress lateral bud growth by inhibiting cytokinin synthesis, and also participates in the feedback regulation of strigolactone synthesis in tomato.

## Introduction

Plant architecture has large impacts on crop yield as in the case of the ‘Green Revolution’ in which a moderate decrease in plant height and increase in tiller number significantly increased grain yields in wheat and rice. Genetic studies on model plants including Arabidopsis (*Arabidopsis thaliana*), rice (*Oryza sativa*), and tomato (*Solanum lycopersicum*) identified mutants defective in shoot branching, angle of the shoot branch or leaves, and inflorescence branching (Wang and Li, 2008). Characterization of the corresponding genes shed light on the molecular basis for designing the ideal plant architecture. In wild-type (WT) tomato that grows indeterminately, an excessive number of lateral shoots is not favorable for flower and fruit development. To ensure fruit production, the lateral shoots are removed manually, which is labor costly and time consuming. Understanding the mechanisms that control the formation of lateral shoots is necessary for reducing the number of lateral shoots and for increasing the efficiency of tomato production.

The development of lateral shoots involves several stages including the initiation of the axillary meristem in the leaf axils, the formation of lateral buds, the activation of lateral buds, and sustained growth of buds into lateral shoots (Wang *et al.*, 2018). Bud formation is mainly determined by transcription factors such as LATERAL SUPPRESSOR (tomato and Arabidopsis) and MONOCULM1 (rice) in the GRAS family (Schumacher *et al.*, 1999; Greb *et al.*, 2003; Li *et al.*, 2003), and BLIND (tomato) and REGULATOR OF AXILLARY MERISTEMS (Arabidopsis) in the MYB family (Schmitz *et al.*, 2002; Müller *et al.*, 2006). The growth of lateral buds is usually inhibited by apical dominance, which has long been thought to be regulated by auxin. However, recent studies showed that sugar availability is the primary determinant of the activation or dormancy of lateral buds (Mason *et al.*, 2014). Sucrose activates bud growth by increasing the expression of *FLOWERING LOCUS T* (*FT*) through the sugar signal trehalose 6-phosphate or by suppressing the expression of *BRANCHED 1* (*BRC1*) (Barbier *et al.*, 2019; Fichtner *et al.*, 2021). *BRC1* encodes a growth-inhibiting transcription factor that is expressed in dormant buds (Aguilar-Martínez *et al.*, 2007; González-Grandío *et al.*, 2017). The transcriptional regulation of *BRC1* by sucrose is shown to be mediated by plant hormones. Sucrose induces the synthesis of cytokinins (CKs) that inhibit the expression of *BRC1* (Salam *et al.*, 2021), but suppresses the signaling of strigolactones (SLs) that promote the expression of *BRC1* (Barbier *et al.*, 2015; Patil *et al.*, 2022). In addition, SLs and CKs show antagonistic interaction in the control of lateral bud growth (Dun *et al.*, 2012), although the underlying mechanism is unclear.

SLs are derived from carotenoids and are plant hormones that inhibit shoot branching (Gomez-Roldan *et al.*, 2008; Umehara *et al.*, 2008). DWARF 27 (D27), CAROTENOID CLEAVAGE DIOXYGENASE 7 (CCD7), CCD8, and MORE AXILLARY GROWTH 1 (MAX1) catalyze the sequential steps in SL synthesis from carotenoids to carlactononic acid (CLA), while cytochrome P450 CYP722s convert CLA to the orobanchol-type SLs (Mashiguchi *et al.*, 2021). SLs are synthesized in roots and translocated to shoots via the ATP-binding cassette transporters (Kretzschmar *et al.*, 2012). Interestingly, a grafting experiment indicated that *de novo* synthesis of SLs in shoots is functional in tomato plants (Visentin *et al.*, 2016). However, SLs are barely detected in shoots, possibly due to the low transcript abundance of SL synthesis genes, and the unique mode of SL perception in which binding of SL by its receptor results in the hydrolysis of SL (Yao *et al.*, 2016). In addition, the expression of SL synthesis genes is negatively feedback regulated by SL signaling (Mashiguchi *et al.*, 2009). SL induces the MORE AXILLARY GROWTH 2 (MAX2)-dependent degradation of DWARF 53 (D53) or D53-like proteins, the repressors of SL signaling (Jiang *et al.*, 2013; Zhou *et al.*, 2013; Wang *et al.*, 2015). D53-like proteins suppress the transcription activity of members of the SQUAMOSA PROMOTER BINDING PROTEIN-LIKE (SPL) family transcription factors, which inhibit tillering and shoot branching in rice and Arabidopsis, respectively (Song *et al.*, 2017; Xie *et al.*, 2020). D53 also directly suppresses the expression of *CYTOKININ OXIDASE 9* (*CKX9*) in rice, leading to significant increases in the CK level and tiller number (Duan *et al.*, 2019).

Transcripts of a subset of SPLs are targeted for cleavage and/or translational repression by miRNA156s (miR156s). The miR156/SPL module is a central hub in regulating the juvenile-to-adult phase transition, plant architecture, flowering, and organ morphology (Wang and Wang, 2015). The allele of the maize *TEOSINTE GLUME ARCHITECTURE1* gene which encodes an SPL protein is a key domestication locus (Dong *et al.*, 2019). Another SPL gene in rice, *IDEAL PLANT ARCHITECTURE 1* (*IPA1*), is a master regulator of plant architecture, and has been considered as a new ‘Green Revolution’ gene (Song *et al.*, 2022). Recently, SPL13 was found to regulate flowering by directly activating the *SINGLE FLOWER TRUSS* gene, an ortholog of *FT*, in tomato (Cui *et al.*, 2020). However, the role of SPLs in shoot branching in tomato is still unclear. Here, we found that SPL13 acted downstream of SLs to suppress shoot branching. SPL13 inhibited lateral bud growth by regulating the CK synthesis gene. In addition, SPL13 participated in feedback regulation of SL synthesis.

## Materials and methods

### Plant materials and growth conditions

The WT tomato (*Solanum lycopersicum* cv. Condine Red) was obtained from the Tomato Genetics Resource Center, UC Davis, CA, USA (https://tgrc.ucdavis.edu) and used to construct the knockout mutants. Seeds were soaked in hot water (50 °C) for 15 min, and then placed in a shaker that was set at a speed of 200 rpm and a temperature of 28 °C to promote germination. When the length of the radicle was 0.5–1 cm, the seeds were sown in the growth substrate composed of peat and vermiculite (3:1 v/v). The seedlings were grown in the plant factory with a controlled environment (light intensity, 200 μmol m^−2^ s^−1^; photoperiod, 12 h/12 h; day/night temperature, 23/20 °C; relative humidity, 70–80%). When the second true leaf fully expanded, the seedlings were transferred to pots containing the same substrate as above, and irrigated 1–2 times a week using Hoagland nutrient solution.

### Construction of the knockout mutants

CRISPR/Cas9 [clustered regularly interspaced palindromic repeats (CRISPR)/CRISPR-associated protein 9] was used to generate *ccd7*, *ccd8*, and *spl13* mutants. The single guide RNA (sgRNA) sequences were designed by the CRISPR-P program (crispr.hzau.edu.cn/CRISPR2/) as follows: *CCD7*, TCCTCCAAAACTCTTGCCAC; *CCD8*, CTTCCTGACATGTTTGATCA; *SPL13*, TCGCCGGCATAAAGTTTGTG.

The gene diagram of *SPL13*, *CCD7*, and *CCD8* showing locations of CRISPR edits is provided in Supplementary Fig. S1.

The synthesized sgRNA sequence was annealed and introduced into the *Bbs*I site of an AtU6–sgRNA–AtUBQ–Cas9 vector. The resulting plasmid was digested by *Hin*dIII and *Kpn*I, and then inserted into the pCAMBIA1301 binary vector digested by the same restriction enzymes. After confirmation by sequencing, the vector containing sgRNA and Cas9 was transformed into *Agrobacterium tumefaciens* strain GV3101. Mutants with defects in the target gene were generated through plant transformation as described previously (Li *et al.*, 2016). T_3_ homozygous lines were used for experiments. Primers used for genotyping are listed in Supplementary Table S1.

### Virus-induced gene silencing

Tobacco rattle virus (TRV)-based vector was used for virus-induced gene silencing (VIGS). To construct the vector, cDNA fragments of target genes were amplified using primers listed in Supplementary Table S1. The VIGS tool of the Sol Genomics Network (https://solgenomics.net/) was used to design the primers. The target site of *IPT1*, *IPT2*, or *IPT4* gene silencing and the location of quantitative PCR (qPCR) primer are shown in Supplementary Fig. S1. Purified PCR products were cloned into the TRV2 vector. After confirmation by sequencing, the plasmids were transformed into *A. tumefaciens* strain GV3101. When the cotyledons fully expanded, the seedlings were infiltrated with a mixture of the *A. tumefaciens* strain carrying a TRV2 derivative and the strain carrying the helper vector TRV1. Plants infiltrated with the empty TRV2 and TRV1 vectors served as controls. The infiltrated plants were kept in the dark for 2 d and then placed in the growth chamber. qPCR was performed to check the gene silencing efficiency. The plants with transcripts of the target gene <40% of the control were used for experiments.

### Chemical treatments and measurement of lateral bud length

Treatment with *rac*-GR24 was performed when plants had four fully expanded leaves. The stock solution of 10 mM *rac*-GR24 was prepared by dissolving 25 mg of *rac*-GR24 (Coolaber, Beijing, China) in 8.38 ml of acetone. For treatment, the stock solution was diluted 1000-fold to make a 10 μM solution. The *rac*-GR24 solution in a volume of 10 μl was applied directly to the lateral buds every day. The length of lateral buds was measured 14 d after *rac*-GR24 treatment using a numeric caliper, perpendicular to the stem.

### In situ hybridization

Tomato lateral buds (~2 mm) were fixed with 4% paraformaldehyde at 4 °C overnight. The samples were washed with phosphate buffer (0.1 M, pH 7.0) three times (5 min each), dehydrated through an ethanol series (30, 50, 70, 80, 90, 95, and 100%) for 30 min at each step, and then treated with 100% ethanol for 1 h. After that, samples were treated through a graded series of xylene in ethanol (25, 50, 75, and 100%), washed with 50% paraffin in xylene at 65 °C for 1 h, incubated with 100% paraffin at 65 °C overnight, embedded in paraffin in molds (MEIKO EC 360), and allowed to solidify at room temperature. Paraffin blocks were cut into 8 μm sections using a Manual Rotary Microtome (Thermo Scientific HM 325) and collected on adhesion microscope slides (MeVid). After dewaxing, *in situ* hybridization was performed using the RNAscope 2.5 HD Detection Kit [Advanced Cell Diagnostics (ACD)] following the manufacturer’s protocol. The *SPL13* probe and a negative control probe for an irrelevant bacterial gene *dapB* were provided by ACD. Slides were imaged on a Zeiss Axio Scope A1 microscope (Zeiss) with a Zeiss Axiocam 503 color camera and Zeiss ZEN imaging software.

### Yeast one-hybrid assay

Yeast one-hybrid (Y1H) assay was performed using the Matchmatch Gold Yeast OneHybrid System (Clontech) according to the manufacturer’s instructions. The *IPT1*, *CCD7*, *MAX1*, *BRC1*, or *SPL13* promoter (2000 bp upstream of the transcriptional start site) was ligated into the pAbAi vector and the full-length coding region of *SPL13* was fused to the pGADT7 vector. The promoter sequences and the *SPL13* coding sequence were amplified using specific primers (Supplementary Table S1). The linearized pAbAi constructs containing the *IPT1*, *CCD7*, *MAX1*, *BRC1*, or *SPL13* promoter were transformed into Y1HGold yeast strain. pGADT7-SPL13 or an empty AD vector was transformed into the modified Y1HGold yeast strain.

### Dual-luciferase assay

*IPT1*, *CCD7*, *MAX1*, *BRC1*, *SPL13*, and *D27* promoters were amplified and ligated into the pGreen II 0800-LUC vector. The full-length coding region of *SPL13* was amplified and fused to the pGreen II 0029 62-SK vector. The primers used to amplify the promoter and *SPL13* coding sequence are listed in Supplementary Table S1. The above constructs were transformed into *A. tumefaciens* strain GV3101. To determine the activity of the promoter as influenced by the transcription factor, a mixture of the *A. tumefaciens* strain carrying the pGreen II 0800-LUC vector or the pGreen II 0029 62-SK vector in a 1:10 ratio was infiltrated into *Nicotiana benthamiana* leaves. Three days later, the promoter activity was determined by the ratio of activity of firefly luciferase (LUC) and the internal reference Renilla luciferase (REN). The LUC and REN activities were measured using a Modulus Luminometer (Promega). The LUC/REN value in the absence of SPL13 protein was set as one.

### Electrophoretic mobility shift assay

EMSA was performed using the Light Shift Chemiluminescent EMSA Kit (Cat. no. 20148; Thermo Fisher Scientific, USA) according to the manufacturer’s instructions. The probes were biotin end-labeled using the Biotin 3ʹ End DNA Labeling kit (Cat. no. 89818; Pierce, USA) and annealed to dsDNA according to the following procedure: 95 °C for 5 min, and then the temperature was decreased from 95 °C to 55 °C in 40 cycles (–1 °C per cycle, 1 cycle min^–1^), 55 °C for 30 min, and then the temperature was decreased from 55 °C to 25 °C by 30 cycles (–1 °C per cycle, 1 cycle min^–1^), and finally 4 °C for 5 min. To produce the recombinant protein of SPL13, the full-length coding sequence of *SPL13* was amplified using the primers listed in Supplementary Table S1. The PCR products were digested with *Bam*HI and *Sac*I, and then ligated into the same sites of the pET-32a vector. The vectors were transformed into *Escherichia coli* strain BL21 (DE3). Expression of the recombinant His-tagged HIS-SPL13 proteins was induced by isopropyl-β-d-1-thiogalactopyranoside. The recombinant proteins were purified according to the instructions of the Novagen pET purification system.

### Measurement of cytokinins and strigolactones

For analysis of CKs, samples (0.2 g) frozen in liquid N_2_ were ground to a fine powder and then ground in 1 ml of ice-cold extraction solution [15:1:4 (v/v/v) methanol:formic acid:water], which was spiked with [^2^H_6_]*N*^6^-isopentenyladenine (D-iP), [^2^H_6_]*N*^6^-isopentenyladenosineriboside (D-iPR), [^2^H_5_]*trans*-zeatin (D-tZ), and [^2^H_5_]*trans*-zeatinriboside (D-tZR). The extracts were kept at –30 °C overnight and centrifuged at 10 000 *g* for 15 min. The supernatants were collected and flowed through a hydrophilic lipophilic balance column (Oasis) which was pre-treated with 1 M formic acid. An aliquot of 0.3 ml of extraction solution was flowed through the column. The liquid was collected and dried under a gaseous N_2_ flush. Samples were dissolved in 1 ml of 1 M formic acid and flowed through a mixed-mode cation (MCX) column (Oasis) which was pre-treated with 1 M formic acid. The column was washed sequentially with 1 ml of 1 M formic acid, 1 ml of methanol, 1 ml of 0.35 M ammonia solution, and 1 ml of 0.35 M ammonia in 60% methanol. The final liquid was dried under gaseous N_2_ flush and dissolved in 200 μl of 1% acetic acid. The mixture was vortexed and then analyzed by Agilent 1290 ultra-HPLC coupled to 6460 triple quadruple mass spectrometers.

Measurement of SLs was performed according to the method of Ruiz-Lozano *et al.* (2016) with modifications. Root samples (0.5 g) were ground in a mortar filled with liquid nitrogen and then extracted with 0.5 ml of 40% acetone (v/v). The homogenates were vortexed for 2 min and centrifuged at 8000 *g* for 5 min. The supernatants were discarded, and the pellets were extracted twice with 0.5 ml of 50% acetone (v/v). The supernatants were filtered through membrane filters (0.22 μm) and then analyzed by Agilent 1290 ultra-HPLC coupled to 6460 triple quadruple mass spectrometers.

### Collection of root exudates

To analyze the SL content in root exudates, the original hydroponic solution was discarded and the roots were washed with ultrapure water. The roots of each intact plant were incubated with 50 ml of freshly prepared Hoagland nutrient solution. The nutrient solution was collected 4 h after light illumination. The nutrient solution of 30 ml was flowed through a MIX column pre-treated with methanol and purified water. The MIX column was sequentially washed with 10, 20, 30, and 40% acetone, and finally the SLs bound to the MIX column were eluted with 6 ml of acetone. The liquid was collected and dried with a gaseous N_2_ flush. The residues were dissolved with 50% acetone and then analyzed by Agilent 1290 ultra-HPLC coupled to 6460 triple quadruple mass spectrometers.

### Gene expression analysis

Total RNA extraction was performed using an RNAprep pure Plant Kit (TIANGEN) according to the operation manual. The first-strand cDNA was synthesized using the ReverTraAce qPCR Reverse Transcription Kit with genome DNA-removing enzyme (Toyobo). qPCR was performed on the LightCycler480 detection system (Roche) using SYBR Super Mix (Takara, RR420A). The conditions included pre-denaturation at 95 °C for 30 s, followed by 40 cycles of 95 °C for 10 s, 60 °C for 30 s, and at 95 °C for 15 s; 60 °C for 60 s; 95 °C for 15 s. Primers for target genes are shown in Supplementary Table S2. Tomato housekeeping genes *ACTIN* and *UBI3* were used as internal reference. Relative expression of target genes was calculated using the comparative 2^−∆∆CT^ method according to Livak and Schmittgen (2001). △△CT=(CT_target gene_–CT_internal reference_)_sample_–(CT_target gene_–CT_internal reference_)_control_.

### Phylogenetic tree construction

The protein sequences of the SPLs in tomato, rice, and Arabidopsis were obtained from the NCBI (http://www.ncbi.nlm.nih.gov/). Multiple sequence alignment was performed using ClustalW, and the output data were saved in MEGA format. The phylogenetic tree was then constructed in MEGA6 software (Sudhir Kumar, Arizona State University, Tempe, AZ, USA) using the Neighbor–Joining (NJ) method with 1000 bootstrap replicates (Tamura *et al.*, 2013).

### Statistical analysis

A completely randomized experimental design was used. Data were subjected to statistical analysis using the SPSS package (SPSS 19.0). The significance of differences between the means was determined by the Student’s *t*-test or Tukey’s test at a level of *P*<0.05.

## Results

### Strigolactones inhibit lateral bud growth by suppressing cytokinin synthesis

**Fig. 1.**
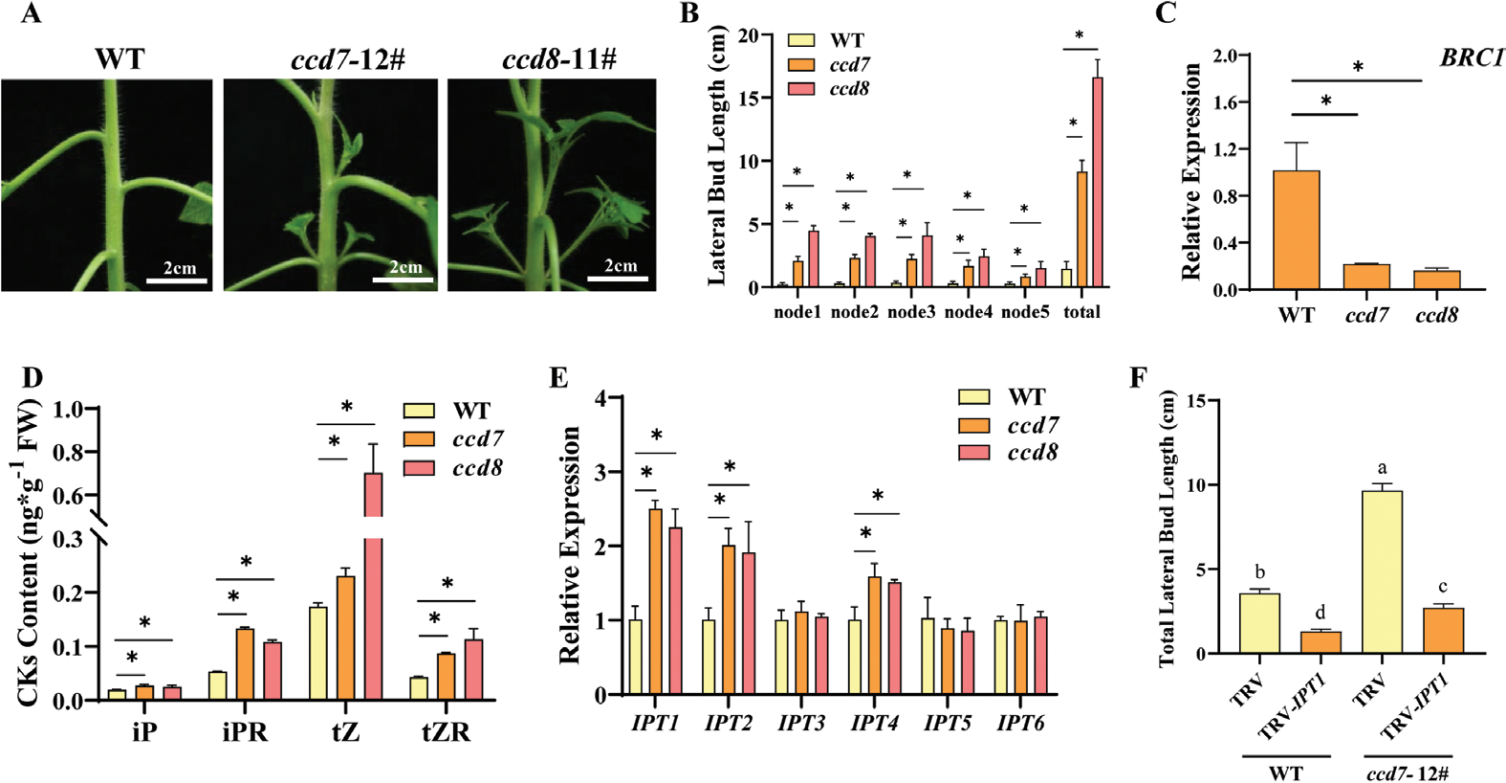
Cytokinin (CK) is involved in strigolactone (SL) regulation of shoot branching. (A) Bud outgrowth phenotypes of *ccd7* and *ccd8* mutants. (B) Lateral bud length of *ccd7* and *ccd8* mutants. (C) qPCR analysis of the relative transcript of *BRC1* in lateral buds. (D) CK content in nodal stems of WT, *ccd7*, and *ccd8* plants. (E) Expression analysis of *IPT1*–*IPT6* in shoots of the WT, and *ccd7* and *ccd8* mutants. (F) Effects of silencing of *IPT1* on the total lateral bud length of WT and *ccd7* plants. Plants in the six-leaf stage were used in the experiment. Values are means of 3–5 biological replicates ±SD. The different letters indicate a significant difference according to Tukey’s test (*P*<0.05), and the asterisks indicate a significant difference according to Student’s *t*-test (*P*<0.05).

Knockdown of the *CCD7* or *CCD8* gene in tomato has been shown to reduce SL levels and to result in increased shoot branching in tomato (Vogel *et al.*, 2010; Kohlen *et al.*, 2012). Here, we generated knockout mutants of tomato *CCD7* and *CCD8* genes using CRISPR/Cas9. Consistent with previous studies, the *ccd7* and *ccd8* mutants showed significantly more lateral buds with expanding leaves as compared with the WT (Fig. 1A). The length of lateral buds in each node was measured acropetally, and was found to be significantly longer than that of the WT in *ccd7* and *ccd8* mutants (Fig. 1B). The total length of lateral buds of *ccd7* and *ccd8* mutants increased by >5-fold and 10-fold, respectively. Consistent with the outgrowth of lateral buds, the expression of *BRC1* was strongly inhibited in lateral buds of *ccd7* and *ccd8* mutants (Fig. 1C).

Next, we studied whether the SL-dependent suppression of shoot branching in tomato was associated with the regulation of CK synthesis. The content of bioactive CK isopentenyladenosine (iP) increased 42.1% and 30.3% and that of iP riboside (iPR) increased 150.2% and 103.0%, respectively, in the shoots of *ccd7* and *ccd8* mutants as compared with the WT (Fig. 1D). Similarly, the contents of *trans*-zeatin (tZ) and tZ riboside (tZR) were significantly increased in *ccd7* and *ccd8* mutants. Analysis of *ISOPENTENYL TRANSFERASE* (*IPT*) gene transcripts encoding the rate-limiting enzyme in CK synthesis indicated that *IPT1*, *IPT2*, and *IPT4* were up-regulated in shoots of *ccd7* and *ccd8* mutants, whereas the expression of *IPT3*, *IPT5*, and *IPT6* was not affected by SL deficiency (Fig. 1E). When the *IPT1* gene was silenced using VIGS, the lateral bud growth was inhibited, whereas silencing of *IPT2* or *IPT4* did not result in apparent changes in bud growth (Supplementary Fig. S2). Notably, the total length of lateral buds of *ccd7* was decreased by 255.8% after *IPT1* gene silencing (Fig. 1F; Supplementary Fig. S3). The results indicated that CK synthesis was involved in SL regulation of shoot branching.

### Inhibition of lateral bud growth by strigolactones is dependent on SPL13

**Fig. 2.**
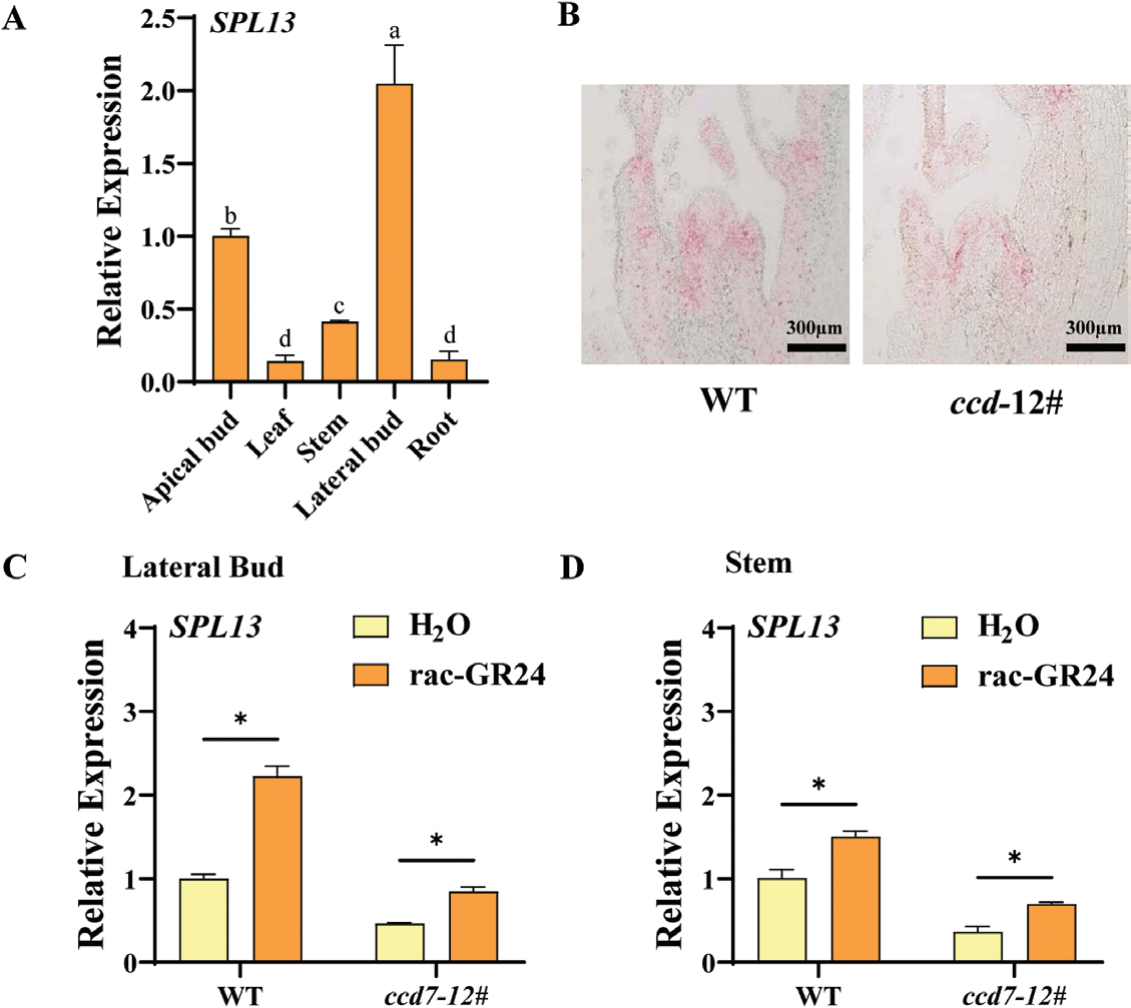
Expression of *SPL13* is regulated by SL. (A) The expression of *SPL13* in tomato apical buds, mature leaves, stems, lateral buds, and roots. (B) *In situ* hybridization of mRNA of *SPL13* in lateral buds. (C and D) Effects of application of *rac*-GR24 on the expression of *SPL13* in lateral buds and stems of the WT and *ccd7* mutant. Plants in the six-leaf stage were used in the experiment. Values are means of three biological replicates ±SD. The different letters indicate a significant difference according to Tukey’s test (*P*<0.05), and the asterisks indicate a significant difference according to Student’s *t*-test (*P*<0.05).

SPLs are critical regulators of shoot branching in rice and Arabidopsis. To study the role of SPLs in the regulation of shoot branching in tomato, the phylogenetic tree that shows the relationship of SPLs in tomato, rice, and Arabidopsis was constructed (Supplementary Fig. S4), and the transcripts of SPL family genes in the lateral buds were compared between the WT and *ccd7* mutant (Supplementary Fig. S5). The results showed that defects in SL synthesis resulted in a 22.1–72.2% decrease in transcript levels of the SPL family genes, among which *SPL13* showed the strongest down-regulation. The pattern of *SPL13* expression showed that the transcript level of *SPL13* was high in lateral buds, moderate in apical buds and stems, and low in mature leaves and roots (Fig. 2A). *In situ* hybridization was carried out to further study the expression pattern of *SPL13*. The mRNA of *SPL13* was detected in the leaf primordia of the lateral buds, and the transcript level of *SPL13* in the *ccd7* mutant was lower than that of the WT (Fig. 2B). In addition, *rac*-GR24 (a synthetic analog of SLs) promoted the expression of *SPL13* in the lateral buds and shoots of the WT, and reversed the inhibition of *SPL13* expression in the *ccd7* mutant (Fig. 2C, D).

**Fig. 3.**
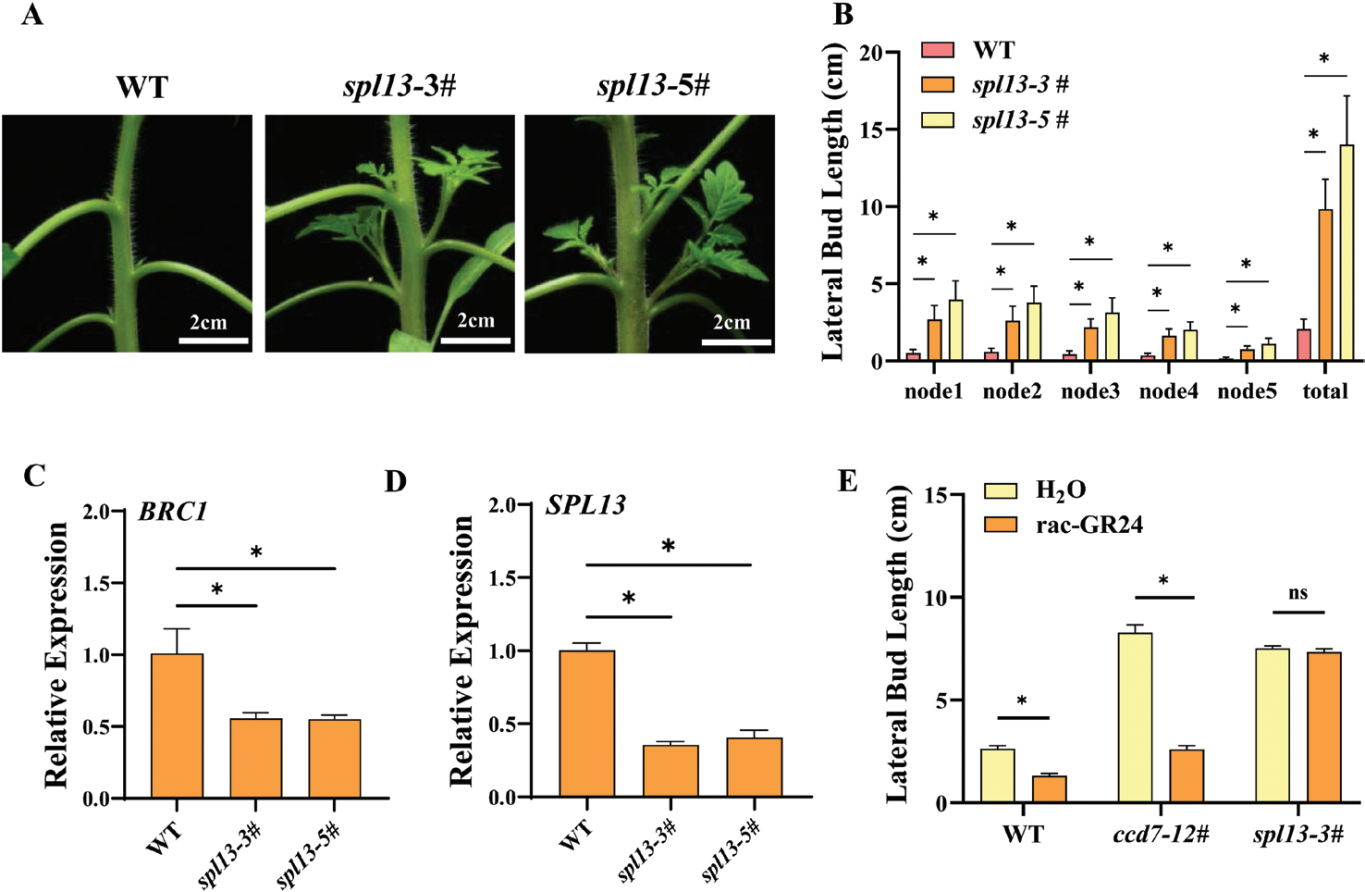
*SPL13* suppresses shoot branching. (A) Bud outgrowth phenotypes of the *spl13* mutant. (B) Lateral bud length of the *spl13* mutant. (C) qPCR analysis of the relative transcript of *BRC1* in lateral buds. (D) qPCR analysis of the relative transcript of *SPL13* in lateral buds of the *spl13* mutant. (E) Effects of application of *rac*-GR24 on the total lateral bud length of *ccd7* and *spl13* mutants. Plants in the six-leaf stage were used in the experiment. Values are means of 3–5 biological replicates ±SD. ns indicates a non-significant difference according to Student’s test (*P*<0.05). The asterisks indicate a significant difference according to Student’s *t*-test (*P*<0.05).

To study whether SPL13 is involved in the SL regulation of shoot branching in tomato, we constructed the knockout mutants of *SPL13* using CRISPR/Cas9 and obtained two lines (*spl13*#3 and *spl13*#5). Similar to *ccd* mutants, the *spl13* mutants showed obvious elongated lateral buds at each node, with the total lateral bud length increasing by 3.74- and 5.75-fold in *spl13*#3 and *spl13*#5, respectively, compared with the WT (Fig. 3A, B). Consistent with the lateral bud growth, the transcript levels of *BRC1* decreased by 44.8% and 45.5% in the two lines, respectively (Fig. 3C). Interestingly, the expression of *SPL13* in lateral buds was strongly inhibited in the *spl13* mutants (Fig. 3D), probably because the transcription of *SPL13* was activated by SPL13 itself (Supplementary Fig. S6). When the plants were treated with *rac*-GR24, the total length of lateral buds in the WT or *ccd7* mutant was significantly inhibited, whereas the lateral bud growth in the *spl13* mutant was not affected (Fig. 3E; Supplementary Fig. S7). All the results indicated that SPL13 acted downstream of SLs to suppress lateral bud growth in tomato.

### SPL13 is involved in strigolactone suppression of cytokinin synthesis

**Fig. 4.**
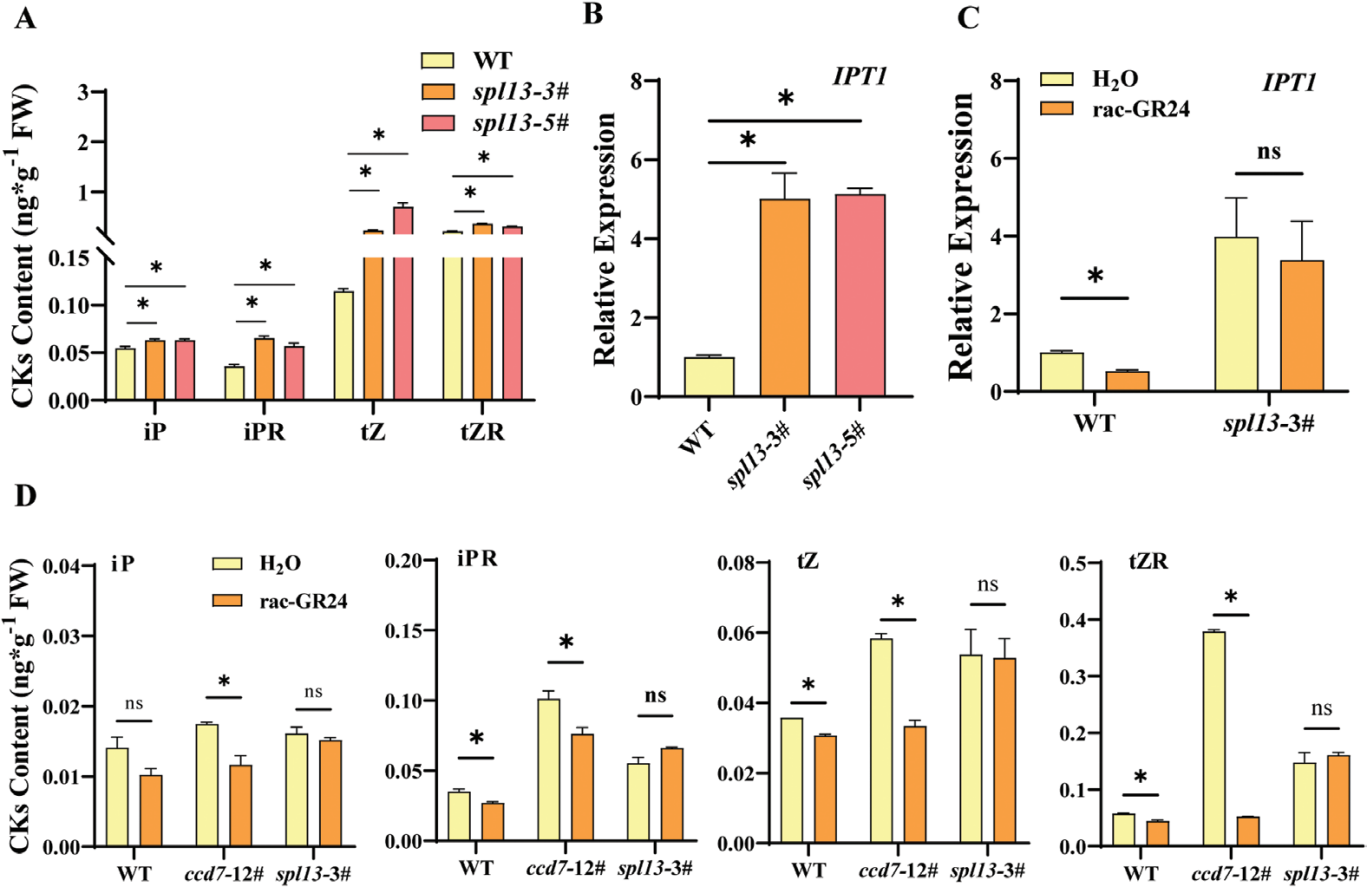
SL suppresses CK synthesis through *SPL13*. (A) CK content in nodal stems of WT and *spl13* plants. (B) Expression analysis of *IPT1* in shoots of the WT and *spl13* mutant. (C) Effects of application of *rac*-GR24 on the expression of *IPT1* in the nodal stems of the WT and *spl13* mutant. (D) Effects of application of *rac*-GR24 on the CK contents of the WT, and *ccd7* and *spl13* mutants. Plants in the six-leaf stage were used in the experiment. Values are means of 3–5 biological replicates ±SD. ns indicates a non-significant difference according to Student’s test (*P*<0.05). The asterisks indicate a significant difference according to Student’s *t*-test (*P*<0.05).

Next, we studied whether SPL13 participated in the regulation of CK synthesis by SLs. Measurement of the CK contents in shoots indicated that the iP, iPR, tZ, and tZR contents were increased by 12.0, 66.7, 207.8, and 393.5%, respectively, in *spl13* mutants compared with the WT (Fig. 4A). The transcript levels of *IPT1* and *IPT2* in shoots of *spl13* mutants were much higher than those of the WT (Fig. 4B; Supplementary Fig. S8), whereas the expression of other members of the IPT gene family in *spl13* mutants shows only slight increases compared with the WT. Furthermore, *rac*-GR24 inhibited the expression of *IPT1* in the WT, but was not able to inhibit the expression of *IPT1* in *spl13* mutants (Fig. 4C). The iP nucleotides can be *trans*-hydroxylated by CYP735A to produce tZ nucleotides, and the CK nucleotides are activated by LONELY GUY (LOG) to produce the bioactive CKs (Werner and Schmulling, 2009). qPCR showed that *LOG3*, *LOG4*, *LOG6*, *CYP735A1*, and *CYP735A2* were up-regulated in *spl13* mutants (Supplementary Fig. S9). In addition, SPL13 regulated the expression of CKXs. The transcript levels of *CKX2* and *CKX6* were lower in *spl13* mutants than in the WT (Supplementary Fig. S10). *rac*-GR24 decreased the iP, iPR, tZ, and tZR content in the shoots of the WT and *ccd7* mutant (Fig. 4D). However, the CK levels in *spl13* mutants were not affected by *rac*-GR24.

### SPL13 inhibits shoot branching by regulating cytokinin synthesis

**Fig. 5.**
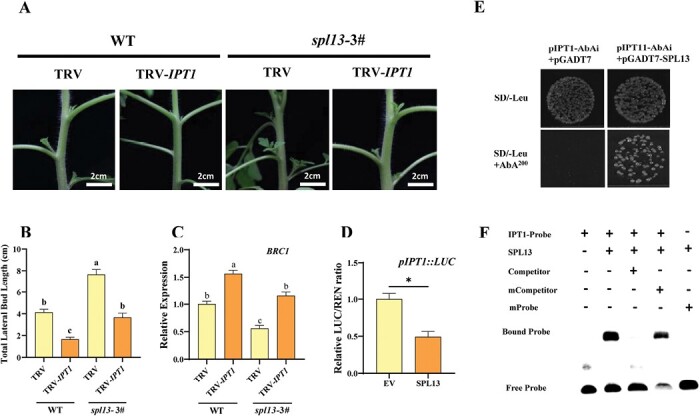
CK synthesis is involved in *SPL13* regulation of shoot branching. (A) Bud outgrowth phenotypes of WT and *spl13* plants after silencing of *IPT1*. (B) Effects of *IPT1* silencing on the total lateral bud length of WT and *spl13* plants. (C) qPCR analysis of the relative transcript of *BRC1* in lateral buds. (D) Dual-luciferase assay for the regulatory effect of SPL13 on the expression of *IPT1*. (E) Y1H analysis of SPL13 binding to the P_*IPT1*_. (F) EMSA analysis of SPL13 binding to the *IPT1*. Plants in the six-leaf stage were used in the experiment. Values are means of 3–5 biological replicates ±SD. The different letters indicate a significant difference according to Tukey’s test (*P*<0.05), and the asterisks indicate a significant difference according to Student’s *t*-test (*P*<0.05).

To further study the role of SPL13 in the regulation of CK synthesis during shoot branching, the CK synthesis gene *IPT1*, whose expression was highest among the IPT family genes in *spl13* mutants, was silenced by VIGS in the *spl13*-3# line. The growth of lateral buds was strongly inhibited by silencing of *IPT1* in the WT and *spl13* mutants (Fig. 5A, B). Accordingly, the transcript levels of *BRC1* in lateral buds were significantly increased in the *IPT1*-silenced WT and *spl13* mutant (Fig. 5C). The transcript of *BRC1* was increased by *rac*-GR24, whereas the expression of *BRC1* was not further enhanced by *rac*-GR24 in *IPT1*-silenced plants (Supplementary Fig. S11). This suggest that *IPT1* is required for SL regulation of *BRC1*. Meanwhile, *rac*-GR24 was not able to promote the expression of *BRC1* in *spl13* mutants (Supplementary Fig. S12C). Consistent with previous findings, the dual-luciferase assay, Y1H assay, and EMSA indicated that SPL13 was able to bind to the promoter of *BRC1* (Supplementary Fig. S13).

Motif analysis of the *IPT1* gene promoter (P_*IPT1*_; sequence 2000 bp upstream of the transcriptional start site) identified five GTAC motifs, the putative SPL-binding sites (Supplementary Fig. S14A). The activity of P_*IPT1*_ decreased by 51% after infiltration of SPL13 protein as shown by the dual-luciferase assay (Fig. 5D). Y1H assay showed that the yeast cells containing the P_*IPT1*_-bait vector and the pGADT7-SPL13 vector grew on the selection medium (Fig. 5E). Furthermore, EMSA confirmed the *in vitro* binding of SPL13 protein to the DNA fragment of P_*IPT1*_ that contained the GTAC motif (Fig. 5F). These results indicated that SPL13 inhibited shoot branching by suppressing the expression of the CK synthesis gene in tomato.

### SPL13 inhibits strigolactone synthesis in tomato

**Fig. 6.**
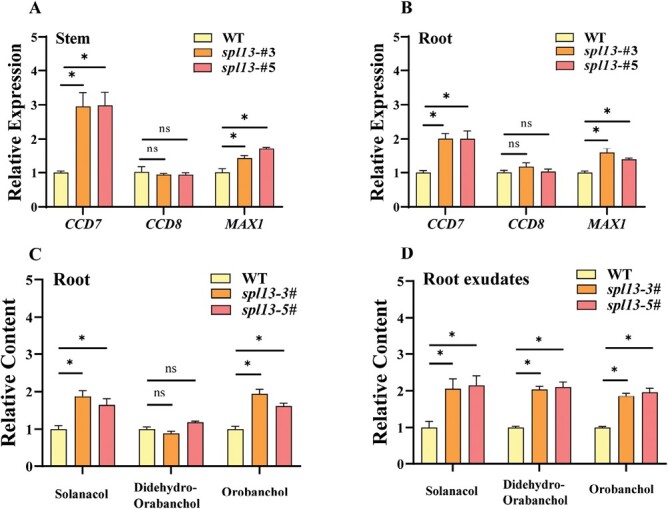
SPL13 inhibits SL synthesis. (A, B) qPCR analysis of relative transcripts of *CCD7*, *CCD8*, and *MAX1* in nodal stems and roots. (C, D) Relative content of orobanchol, didehydro-orobanchol, and solanacol in root extracts and root exudates. Plants in the six-leaf stage were used in the experiment. Values are means of three biological replicates ±SD. ns indicates a non-significant difference according to Student’s test (*P*<0.05). The asterisks indicate a significant difference according to Student’s *t*-test (*P*<0.05).

To further study the relationship between SLs and SPL13, the SL synthesis in *spl13* mutants was analyzed. Compared with the WT, the transcript levels of *CCD7* and *MAX1* in the roots and shoots of *spl13* mutants increased by 38.1–200.7% (Fig. 6A, B). However, no significant difference in the expression of *CCD8* was found between the WT and *spl13* mutants. The transcript levels of *CCD7* and *MAX1* decreased by 51.1% and 36.8 %, respectively, in the WT after *rac*-GR24 treatment, whereas *rac*-GR24-induced inhibition of *CCD7* and *MAX1* expression was not obvious in *spl13* mutants (Supplementary Fig. S12A, B). *CYP722C*, *CYP712G1*, and *D27* also function in SL synthesis. qPCR results showed that the expression of *CYP722C* and *CYP712G1* did not change significantly, whereas the expression of *D27* was significantly increased in roots of *spl13* mutants. Dual-luciferase assay showed that SPL13 can suppress the promoter activity of *D27* (Supplementary Fig. S15). Orobanchol, didehydro-orobanchol, and solanacol were detected in the root extracts and exudates. The relative levels of orobanchol and solanacol in root extracts of *spl13* mutants were higher than those of the WT, whereas the relative level of all three types of SLs increased in the root exudates of *spl13* mutants as compared with the WT (Fig. 6C, D). The *ccd7* shoot branching phenotype was reversed when *ccd7* scions were grafted on the *spl13* rootstocks (Supplementary Fig. S16), further indicating sufficient amounts of SLs in the roots of the *spl13* mutant.

**Fig. 7.**
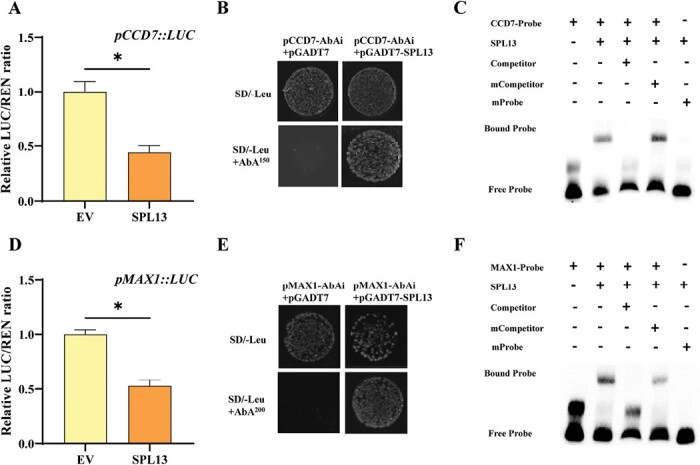
SPL13 inhibits the transcription of SL synthesis genes. (A, D) Dual-luciferase assay for the regulatory effect of SPL13 on the expression of *CCD7* and *MAX1*. (B, E) Y1H analysis of SPL13 binding to the P_*CCD7*_ and P_*MAX1*_. (C, F) EMSA analysis of SPL13 binding to *CCD7* and *MAX1*. Values are means of four biological replicates ±SD. The asterisks indicate a significant difference according to Student’s *t*-test (*P*<0.05).

Dual-luciferase assay showed that the activity of the *CCD7* and *MAX1* promoters, which contained two and four GTAC motifs, respectively (Supplementary Fig. S14B, C), was inhibited by 55.9% and 47.2%, as a result of SPL13 infiltration (Fig. 7A, D). The Y1H assay also showed interaction between SPL13 and the promoters of *CCD7* or *MAX1* (Fig. 7B, E). In addition, EMSA confirmed the binding of SPL13 protein to the GTAC motif in the promoters of *CCD7* and *MAX1* (Fig. 7C, F). All the results suggested that SPL13 was a transcriptional repressor of *CCD7* and *MAX1*, and was involved in the feedback regulation of SLs synthesis.

## Discussion

### SPL13 mediates strigolactone to regulate shoot branching in tomato

Molecular genetic studies in model plants have established the pathways of SL synthesis (Mashiguchi *et al.*, 2021). Previous studies have shown the inverse relationship between SL levels and the number of lateral shoots in *CCD7*- or *CCD8-*silenced tomato plants and demonstrated that *CCD7* and *CCD8* are essential for SL synthesis in tomato (Vogel *et al.*, 2010; Kohlen *et al.*, 2012). However, the mechanism by which SLs regulate shoot branching in tomato is unclear. In Arabidopsis or rice, D53 or D53-like protein which is targeted for protein degradation in SL signaling suppress the function of SPL transcription factors which suppress shoot branching or tillering (Song *et al.*, 2017; Xie *et al.*, 2020). The miR156–SPL14 module was also shown to inhibit tillering independently of SLs in rice (Luo *et al.*, 2012). A previous study showed that the tomato miR156a-targeted *SPL13* regulates inflorescence development (Cui *et al.*, 2020). However, the role of SPL13 in the suppression of shoot branching by SLs in tomato was unclear. By analyzing the transcript levels of SPL family genes in SL-deficient *ccd7* mutants, the expression of *SPL13* was found to be the most inhibited (Supplementary Fig. S5). The transcripts of *SPL13* were most abundant in lateral buds (Fig. 2A), and knockout of *SPL13* promoted the outgrowth of lateral buds as compared with the WT (Fig. 3A, B), indicating that SPL13 is an important regulator of lateral bud growth. Importantly, the lateral bud growth of *ccd7* was inhibited when the SL deficiency was complemented by exogenous *rac*-GR24, whereas the growth of lateral buds in *spl13* mutants was insensitive to *rac*-GR24. In addition, the *spl13* mutants contained higher levels of SLs in root extracts and exudates, which was attributable to an increase in the expression of SL synthesis genes (Fig. 6). The results provide evidence supporting that SPL13 is involved in SL regulation of shoot branching, and participates in the feedback regulation of SL synthesis in tomato. Interestingly, the *ccd7* shoot branching phenotype was reversed when *ccd7* scions were grafted on the WT or *spl13* rootstocks (Supplementary Fig. S16). Long-distance SL transport from roots to shoots was shown to be mediated by a short-distance transporter (Kretzschmar *et al.*, 2012; Shiratake *et al.*, 2019), instead of the xylem sap flow. The grafting experiment further excludes the possibility that the shoot branching phenotypes of *spl13* mutants is related to SL transport.

At present, the mechanism by which SPL13 is controlled by the upstream regulator of SL signaling is unclear in tomato. We found that the *SPL13* gene was transcriptionally self-regulated. The promoter activity of *SPL13* (P_*SPL13*_) increased after infiltration of SPL13 protein, as shown by the dual-luciferase assay (Supplementary Fig. S6A). The Y1H assay showed that the yeast cells containing the P_*SPL13*_-bait vector and the pGADT7-SPL13 vector grew on the selection medium (Supplementary Fig. S6B). SPL13 protein is necessary for the transcriptional induction of *SPL13* by SLs (Fig. 3E). Recently, a D53-like gene was identified in *Pisum sativum* and was found to act downstream of SLs to regulate lateral bud growth (Kerr *et al.*, 2021). If SLs regulate shoot branching in tomato through the conserved signaling pathway, the putative D53-like protein is the most likely regulator of SPL13. The upstream SL signaling will release the suppression of SPL13 by the D53-like protein, leading to the activation of *SPL13* transcription. This explains why the *SPL13* gene was regulated by SLs at the transcriptional level (Fig. 2).

### Cytokinin synthesis participates in the strigolactone inhibition of shoot branching

SLs and CKs have antagonistic effects on lateral bud growth (Dun *et al*, 2012). However, the role of CKs in the SL regulation of lateral bud growth remains ambiguous. CK levels in the xylem sap were reduced in the SL synthesis or signaling mutants, possibly due to a feedback mechanism (Foo *et al.*, 2007). However, *IPT1*, the key gene involved in local CK synthesis during release of apical dominance (Tanaka *et al.*, 2006), was up-regulated in the SL-related mutants in pea (*P. sativum*) (Dun *et al.*, 2012). CK levels were also found to increase in shoots of the SL synthesis mutant in pea (Young *et al.*, 2014). In addition, GR24 down-regulated the expression of *IPT3* in chrysanthemum (Chen *et al.*, 2013). In this study, the tomato *ccd* mutants showed a significantly higher level of CKs in shoots compared with the WT. The increase in CK content was related to the up-regulation of the *IPT1* gene, whereas silencing of *IPT1* strongly inhibited the outgrowth of lateral buds in *ccd7* mutants (Fig. 1E). Local CK synthesis is required for the initial activation of lateral buds (Tanaka *et al.*, 2006; Dun *et al*, 2012). Additionally, CKs promote vascular development (Fu *et al.*, 2021) which is likely to be involved in the polar auxin transport and sugar transport during the activation of lateral buds (Waldie and Leyser, 2018; Barbier *et al.*, 2019). CK also regulates SL signaling through enhancing the expression of *D53-like* genes, which probably regulate the expression of downstream genes directly or by suppressing the transcriptional activity of multiple SPLs (Kerr *et al.*, 2021). Combining the results of this study, it is clear that suppressing CK synthesis is involved in the SL inhibition of bud growth.

### The function of SPL13 is intimately linked to cytokinin synthesis

**Fig. 8.**
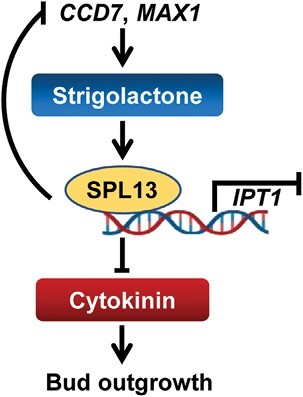
The model depicts the mechanism of action of *SPL13* in suppressing shoot branching in tomato plants.

SLs suppress CK levels by promoting the expression of a CK degradation gene in a D53-dependent manner in rice (Duan *et al.*, 2019). Here, we propose a model whereby SPL13 mediated SLs to suppress local CK synthesis by inhibiting the expression of *IPT1* (Fig. 8). Exogenous SL inhibited local CK synthesis in the WT, but failed to regulate CK synthesis in the *spl13* mutants. Mutation of the SL-regulated *SPL13* resulted in an increase in *IPT1* transcripts and elevated CK levels in shoots (Fig. 4A, B). Furthermore, SPL13 directly bound to the promoter of the *IPT1* gene, whereas silencing of *IPT1* suppressed lateral bud growth in *spl13* mutants (Fig. 5). The evidence strongly supports that SLs inhibit CK synthesis through SPL13.

Silencing of *IPT1* reversed the inhibition of *BRC1* expression in the *spl13* mutant (Fig. 5C). In addition, GR24 increased the expression of *BRC1* in the TRV control, but did not further enhance the expression of *BRC1* in *IPT1*-silenced plants (Supplementary Fig. S11). The results suggest that SL and SPL13 are regulating *BRC1* expression indirectly through CKs. However, dual-luciferase and Y1H assays indicated that SPL13 can bind to the promoter of *BRC1*, consistent with previous studies showing that IPA1/SPLs directly regulate *BRC1* expression (Lu *et al.*, 2013; Xie *et al.*, 2020). We propose that SPL13 has dual functions in directly regulating *BRC1* expression and CK synthesis, while both mechanisms are involved in SL regulation of bud outgrowth. Recently, *D53-like* genes, the suppressors of SL signaling, were found to be induced by CK signaling (Kerr *et al.*, 2021). It is likely that CK participates in the SPL13 regulation of *BRC1* expression through impacts on SL signaling. In the absence of sufficient CKs, low levels of D53-like allow transcriptional activation of the *BRC1* gene by multiple SPL factors. Alternatively, CK signaling may directly regulate the expression of *BRC1*.

We also found that tomato SPL13 is not homologous to IPA1/SPL14 in rice and SPL9/15 in Arabidopsis (Supplementary Fig. S4). Combined with the roles of SPL13 in *BRC1* transcription and CK synthesis, our results suggest that the mode of action of SPL13 is distinct from those of the classic SPLs. Recently, the SPL transcription factor LIGULELESS1 was shown to regulate leaf angle in maize by activating the transcription of *RAVL1*, which encodes a B3-domain transcription factor acting upstream of brassinosteroids (Tian *et al.*, 2019). SPL8 was also found to promote lamina joint development by regulating auxin signaling and brassinosteroid biosynthesis in wheat (Liu *et al.*, 2019). These results indicate the functional diversity of SPL factors, and emphasize that SPL-dependent regulation of hormone synthesis and/or signaling underlies the shaping of plant architecture in different crops.

The pattern of *SPL13* expression showed that the *SPL13* transcripts were abundant in lateral buds (Fig. 2A), suggesting that SPL13 may directly control the genes related to leaf differentiation and growth of the buds. A previous study showed that the lateral buds of mutants with defects in CK synthesis still showed a response to a decrease in apical dominance (Müller *et al.*, 2015). SLs regulate lateral bud dormancy downstream of auxin (Brewer *et al.*, 2009). It is likely that SLs also regulate shoot branching through a pathway independent of CKs. Indeed, the length of lateral buds in *ccd7* mutants was still longer than that of the WT after silencing of the *IPT1* gene (Fig. 1F; Supplementary Fig. S3). It is likely that SPL13-dependent transcription of growth-inhibiting genes in buds plays a role in SL-mediated bud dormancy. However, the detailed mechanism needs in-depth studies in the future. Importantly, the allele of the *SPL13* gene that results in compact shoot architectures suitable for growing at high density should be identified in tomatoes. Additionally, the advance in gene editing technology is expected to aid in fine regulation of *SPL13* expression in tomato plants, leading to the optimization of vegetative and reproductive growth, and ultimately a boost in fruit yields.

## Supplementary data

The following supplementary data are available at *JXB* online.

Fig. S1. Gene diagram.

Fig. S2. *IPT1* is important for the regulation of lateral bud growth.

Fig. S3. Bud outgrowth phenotypes of the WT and *ccd7* mutant after silencing of the CK synthesis gene *IPT1*.

Fig. S4. Phylogenetic tree that shows the relationship of SPLs in tomato, rice, and Arabidopsis.

Fig. S5. Expression analysis of SPL family genes in the lateral buds of the WT and *ccd7* mutants.

Fig. S6. The expression of *SPL13* undergoes self-regulation.

Fig. S7. Bud outgrowth phenotypes of the *ccd7* and *spl13* mutants after application of *rac*-GR24.

Fig. S8. Expression analysis of *IPT2*–*IPT6* in shoots of the WT and *spl13* mutants.

Fig. S9. Expression analysis of *LOG1*–*LOG8*, *CYP735A1*, and *CYP735A2* in shoots of the WT and *spl13* mutants.

Fig. S10. Expression analysis of *CKX1*–*CKX7* in shoots of the WT and *spl13* mutants.

Fig. S11. qPCR analysis of the relative transcript of *BRC1* in lateral buds.

Fig. S12. Effects of application of *rac*-GR24 on the expression of *CCD7* and *MAX1* in shoots and *BRC1* in lateral buds of the WT and *spl13* mutant.

Fig. S13. SPL13 promotes the transcription of *BRC1*.

Fig. S14. Identification of the SPL-binding motif in the promoters of *IPT1*, *CCD7*, *MAX1*, *BRC1*, *CCD8*, and *D27*.

Fig. S15. Expression analysis of *CYP722C*, *CYP712G1*, and *D27* in roots of the WT and *spl13* mutants.

Fig. S16. Effects of grafting on the lateral bud outgrowth.

Table. S1. Primers used for vector construction.

Table. S2. Primers used for qPCR analysis.

erad303_suppl_Supplementary_Figures_S1-S16_Tables_S1-S2

## Data Availability

All data supporting the findings of this study are available within the paper and within its supplementary data published online.
