# Supplementary material for: The transcription factor SPL13 mediates strigolactone suppression of shoot branching by inhibiting cytokinin synthesis in Solanum lycopersicum
Source: J Exp Bot. 2023 Jul 28;74(18):5722–35. doi: 10.1093/jxb/erad303 (PMC10540736; doi:10.1093/jxb/erad303)
Supplement: erad303_suppl_Supplementary_Figures_S1-S16_Tables_S1-S2 [file erad303_suppl_supplementary_figures_s1-s16_tables_s1-s2.pdf]

Supplemental Fig. S1

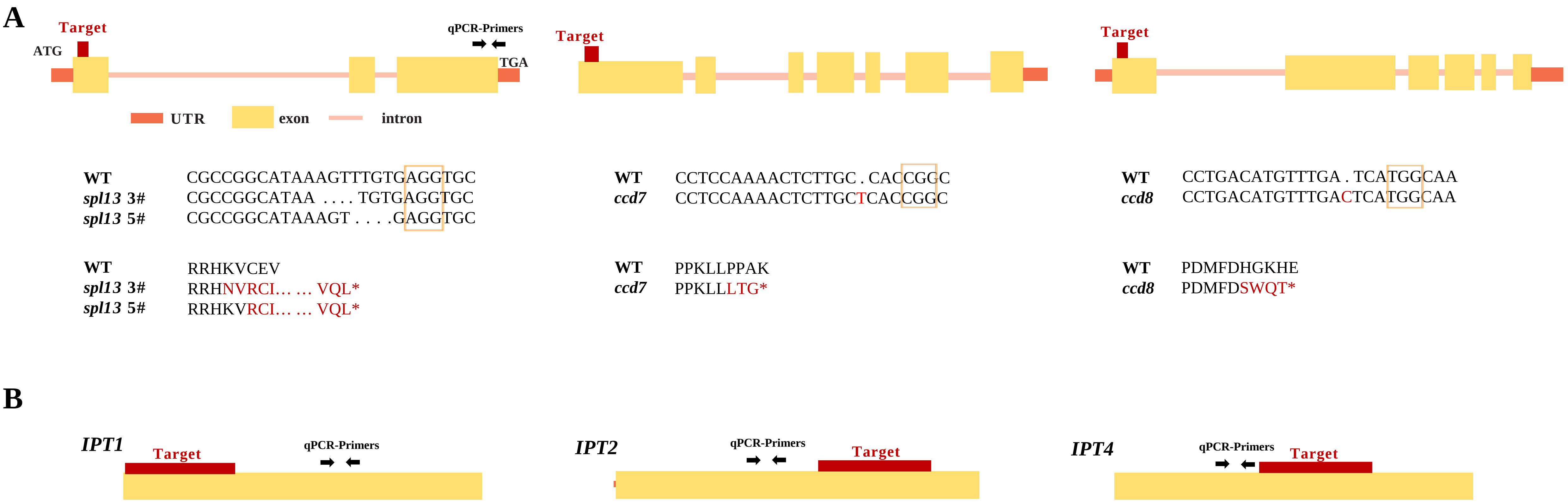

Supplemental Fig. S1. Gene diagram. (A)The locations of CRISPR/Cas9 target and primers for measuring gene expression. (B)The VIGS target in the *IPT1*, *IPT2* and *IPT4* gene.

Supplemental Fig. S2

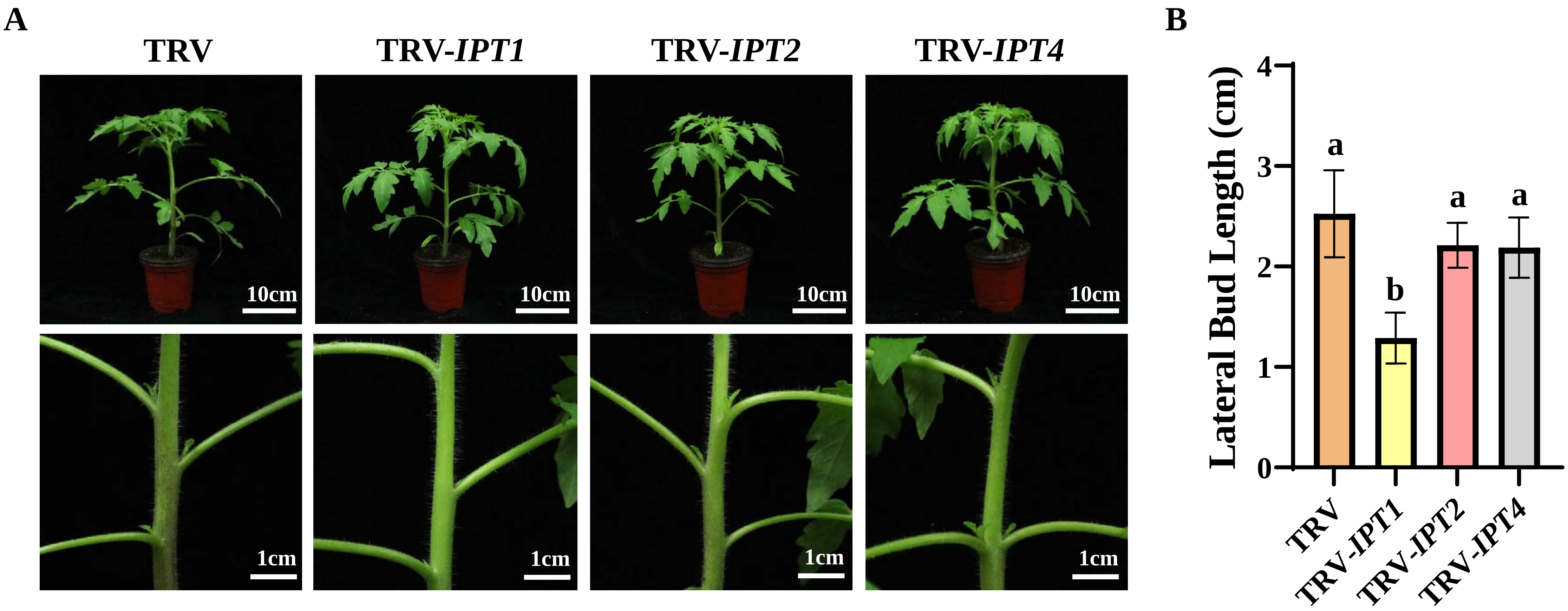

Supplemental Fig. S2. *IPT1* is important for the regulation of lateral bud growth. (A) Bud outgrowth phenotypes of WT after silencing of *IPT1*, *IPT2* and *IPT4*. (B) Total lateral bud length. Plants in the 6-leaf stage were used in the experiment. Values are means of three biological replicates ± SD. The different letters indicate significant difference according to Tukey's test ($p < 0.05$).

Supplemental Fig. S3

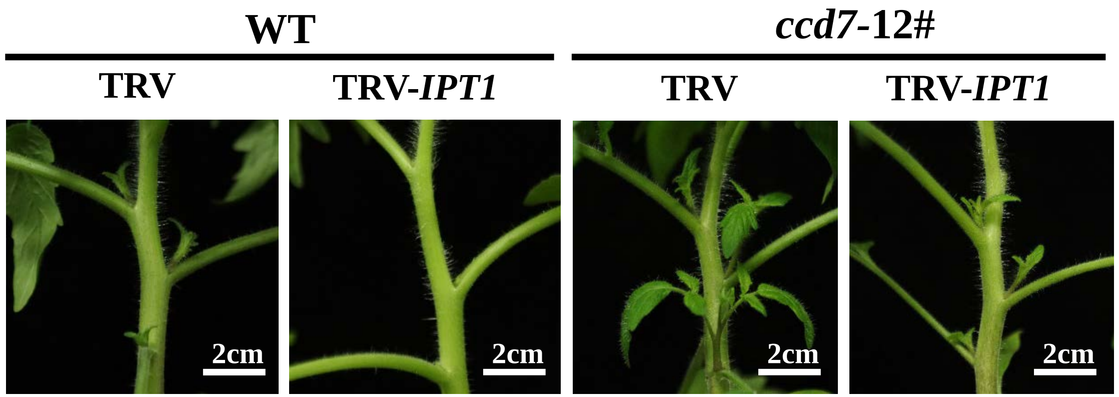

Supplemental Fig. S3. Bud outgrowth phenotypes of WT and *ccd7* mutant after silencing of CK synthesis gene *IPT1*. Plants in the 6-leaf stage were used in the experiment.

Supplemental Fig. S4

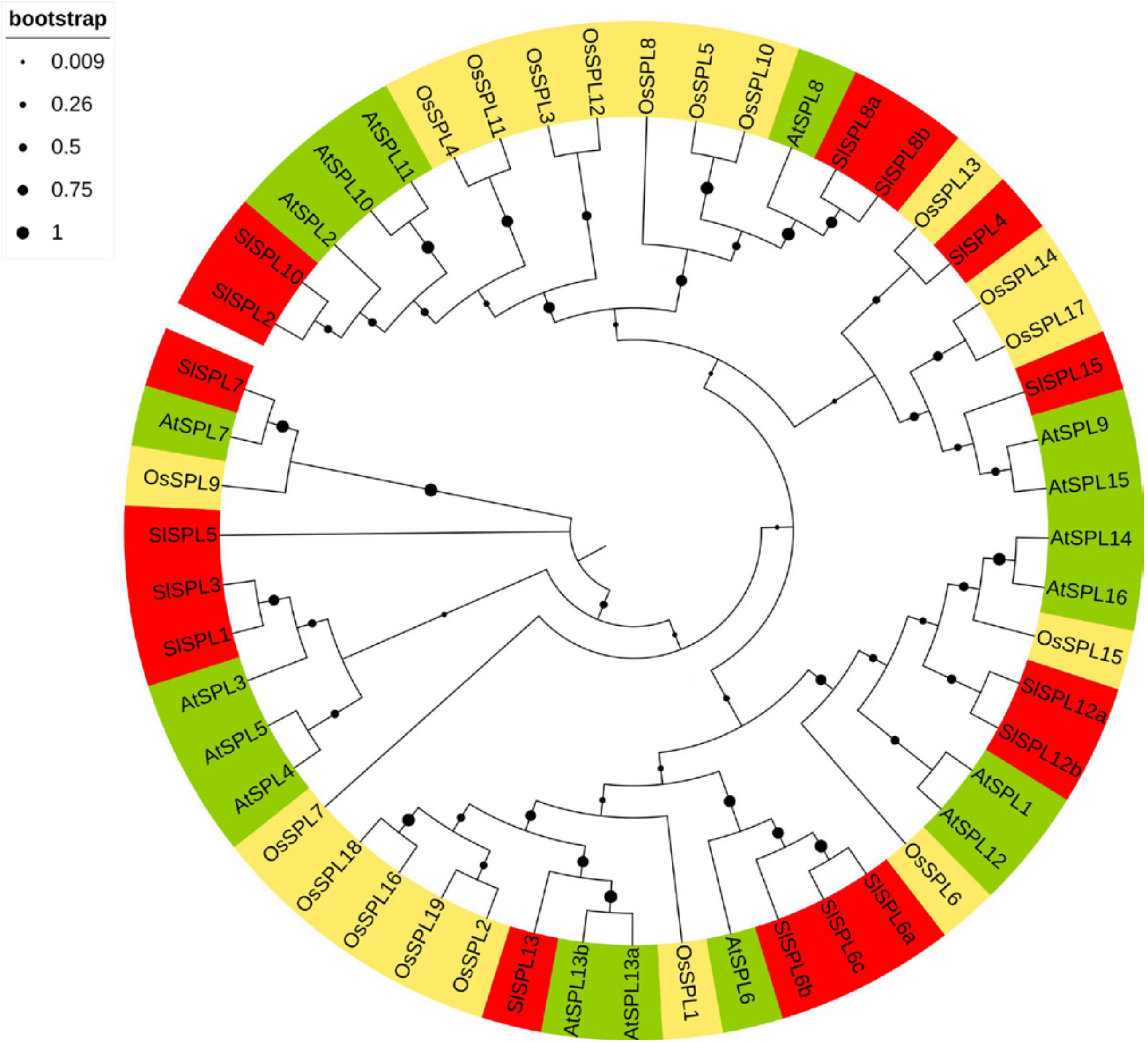

Supplemental Fig. S4. Phylogenetic tree that shows the relationship of SPLs in tomato, rice and Arabidopsis.

Supplemental Fig. S5

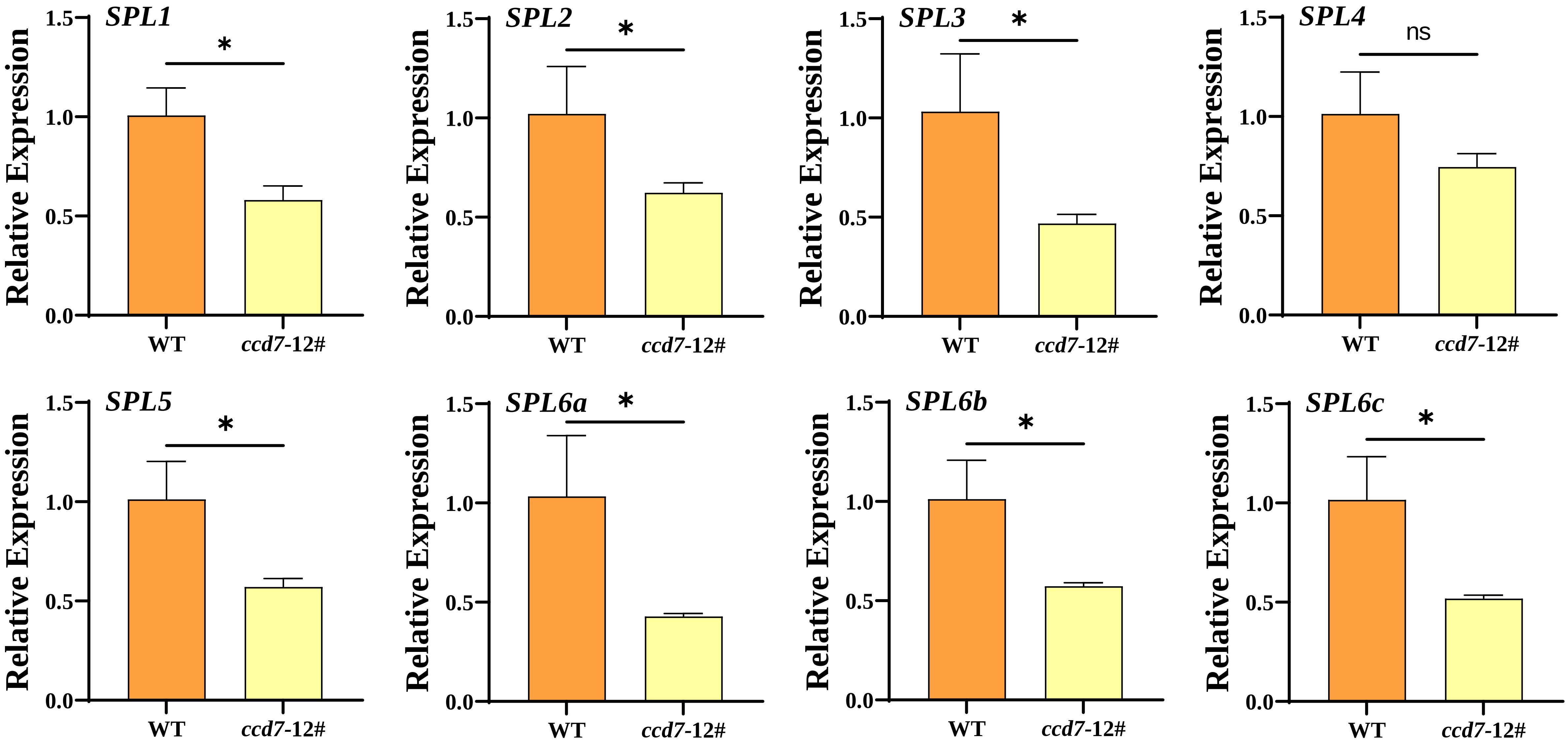

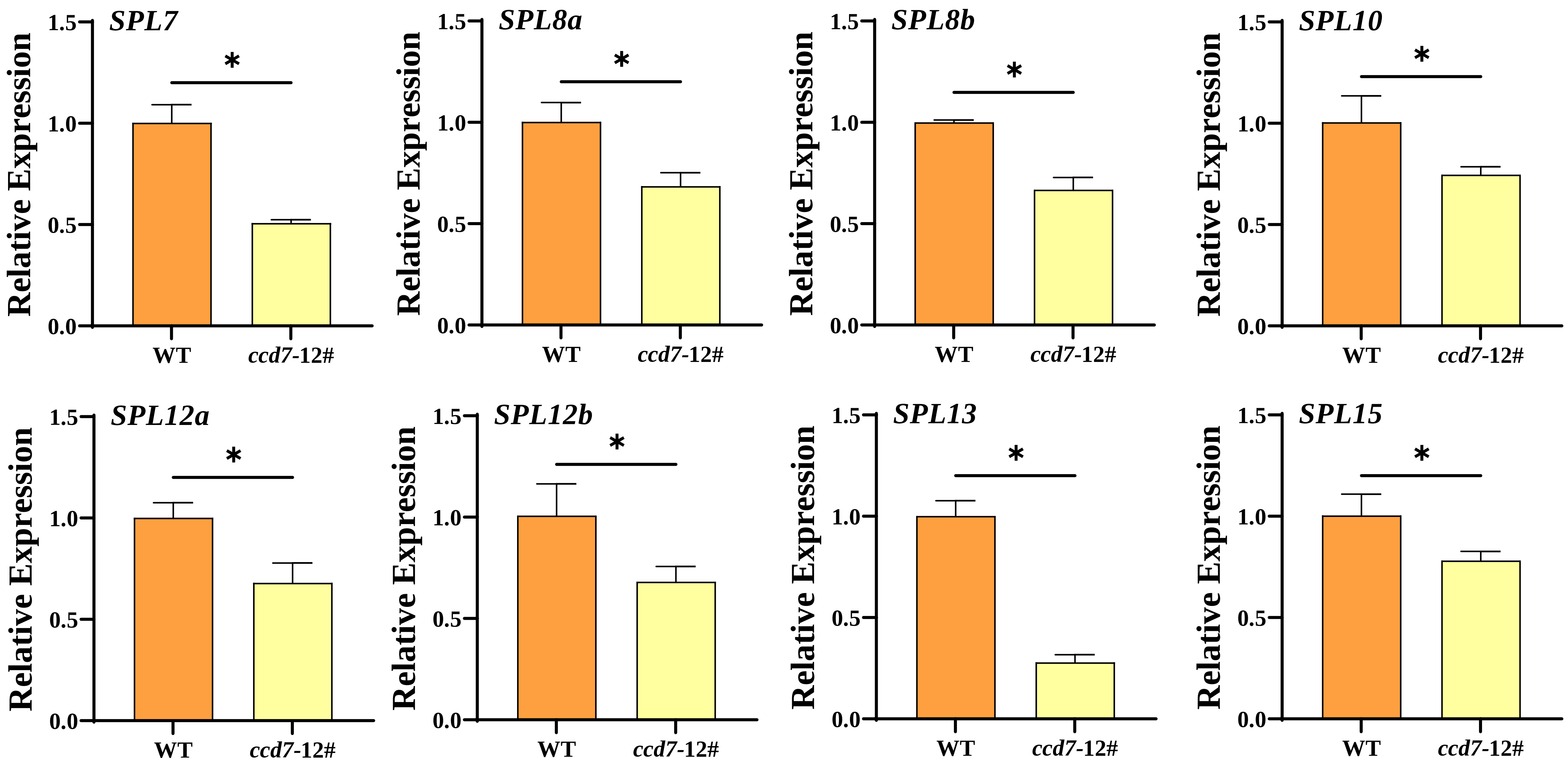

Supplemental Fig. S5. Expression analysis of SPL family genes in the lateral buds of WT and *ccd7* mutants. Plants in the 6-leaf stage were used in the experiment. Values are means of three biological replicates ± SD. The ns indicate insignificant difference according to Student's test ($p < 0.05$). The asterisks indicate significant difference according to Student's test ($p < 0.05$).

Supplemental Fig. S6

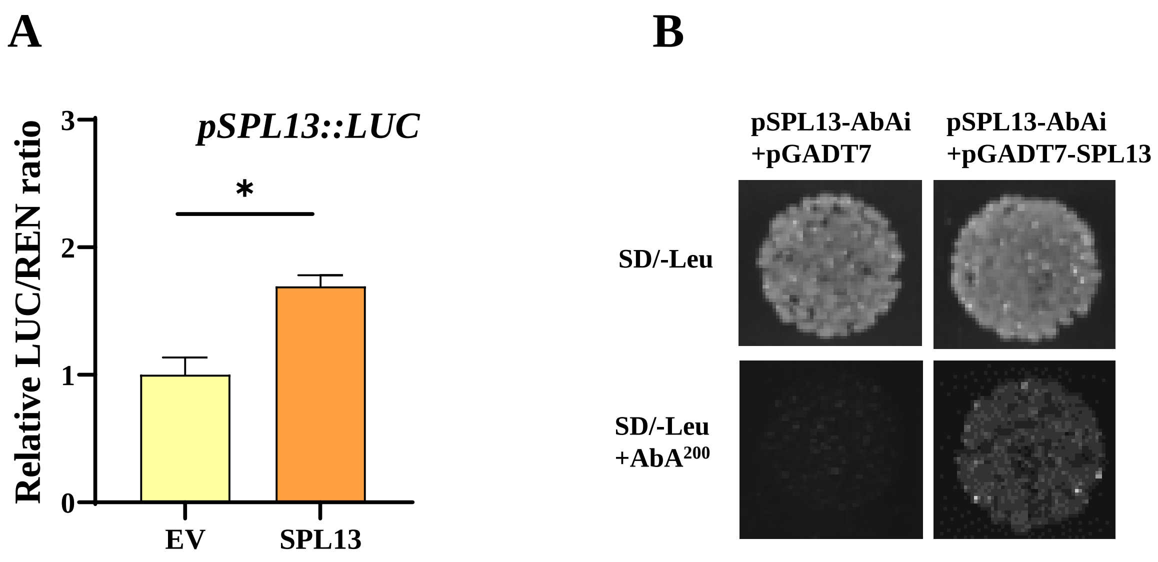

Supplemental Fig. S6. The expression of *SPL13* undergoes self-regulation. (A) Dual-luciferase assay for the regulation of *SPL13* expression by SPL13 protein. (B) Y1H analysis of SPL13 binding to the P_{SPL13}. Values are means of six biological replicates ± SD. The asterisks indicate significant difference according to Student's test ($p < 0.05$).

Supplemental Fig. S7

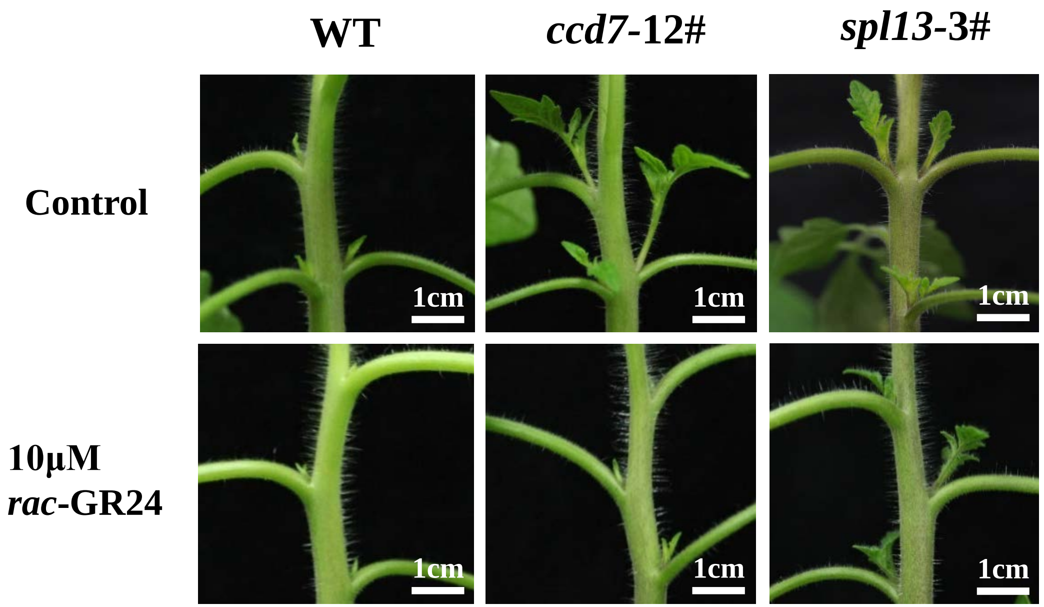

Supplemental Fig. S7. Bud outgrowth phenotypes of the *ccd7* and *spl13* mutants after application of *rac*-GR24. Plants in the 6-leaf stage were used in the experiment.

Supplemental Fig. S8

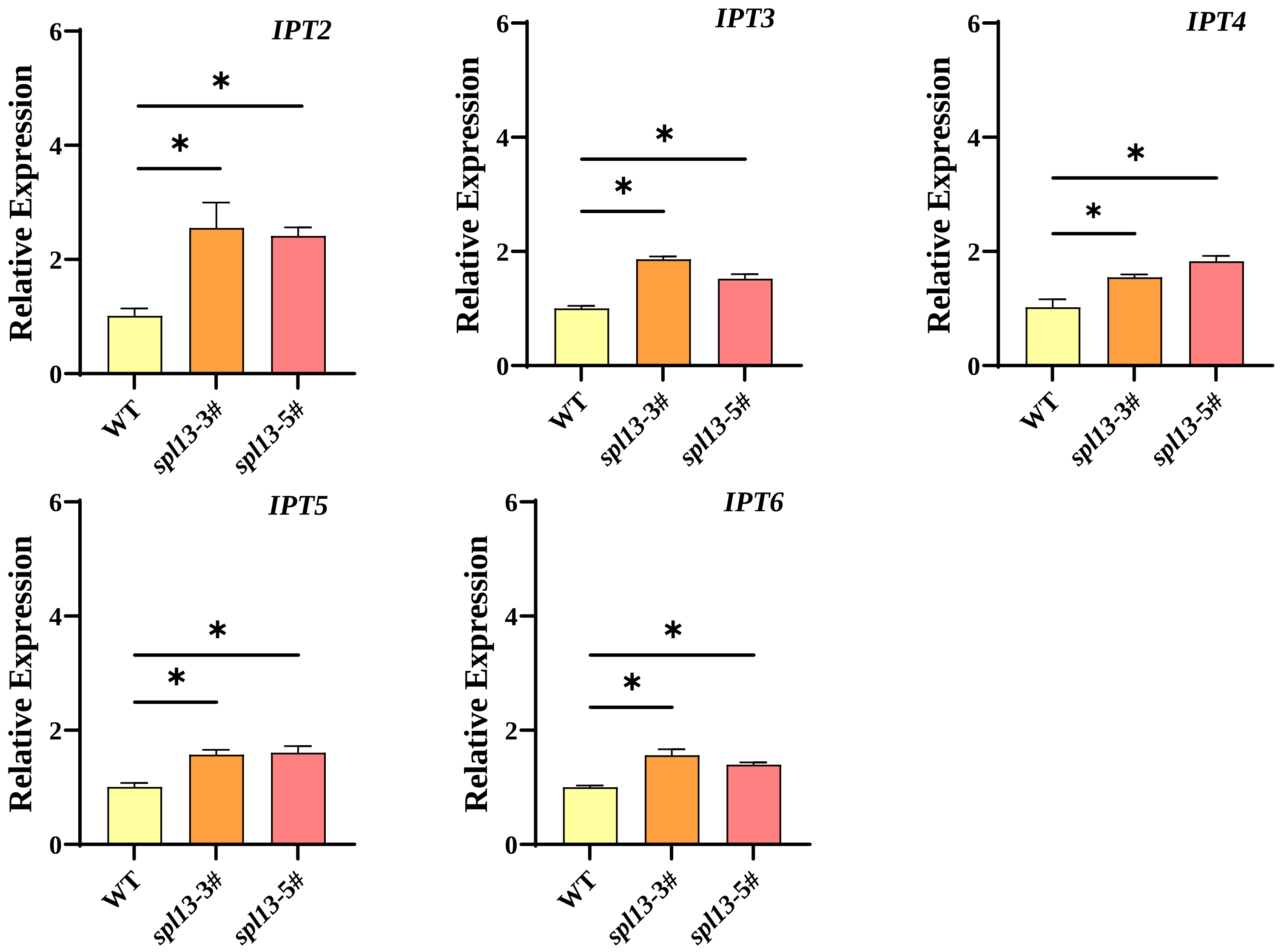

Supplemental Fig. S8. Expression analysis of *IPT2*-*IPT6* in shoots of WT and *spl13* mutants. Plants in the 6-leaf stage were used in the experiment. Values are means of three biological replicates ± SD. The asterisks indicate significant difference according to Student's test ($p < 0.05$).

Supplemental Fig. S9

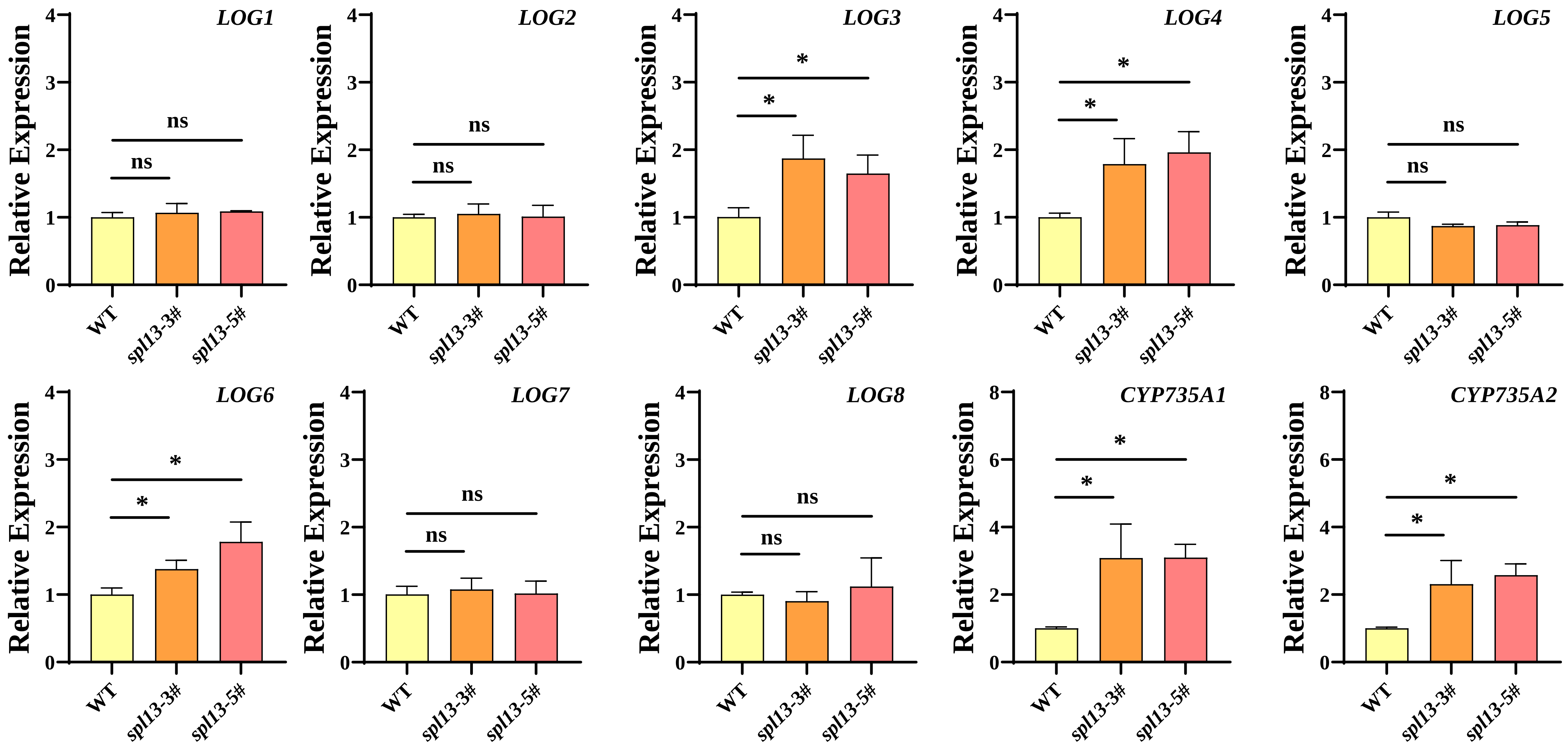

Supplemental Fig. S9. Expression analysis of *LOG1-LOG8*, *CYP735A1* and *CYP735A2* in shoots of WT and *spl13* mutants. Plants in the 6-leaf stage were used in the experiment. Values are means of three biological replicates ± SD. The ns indicate insignificant difference according to Student's test ($p < 0.05$). The asterisks indicate significant difference according to Student's test ($p < 0.05$).

Supplemental Fig. S10

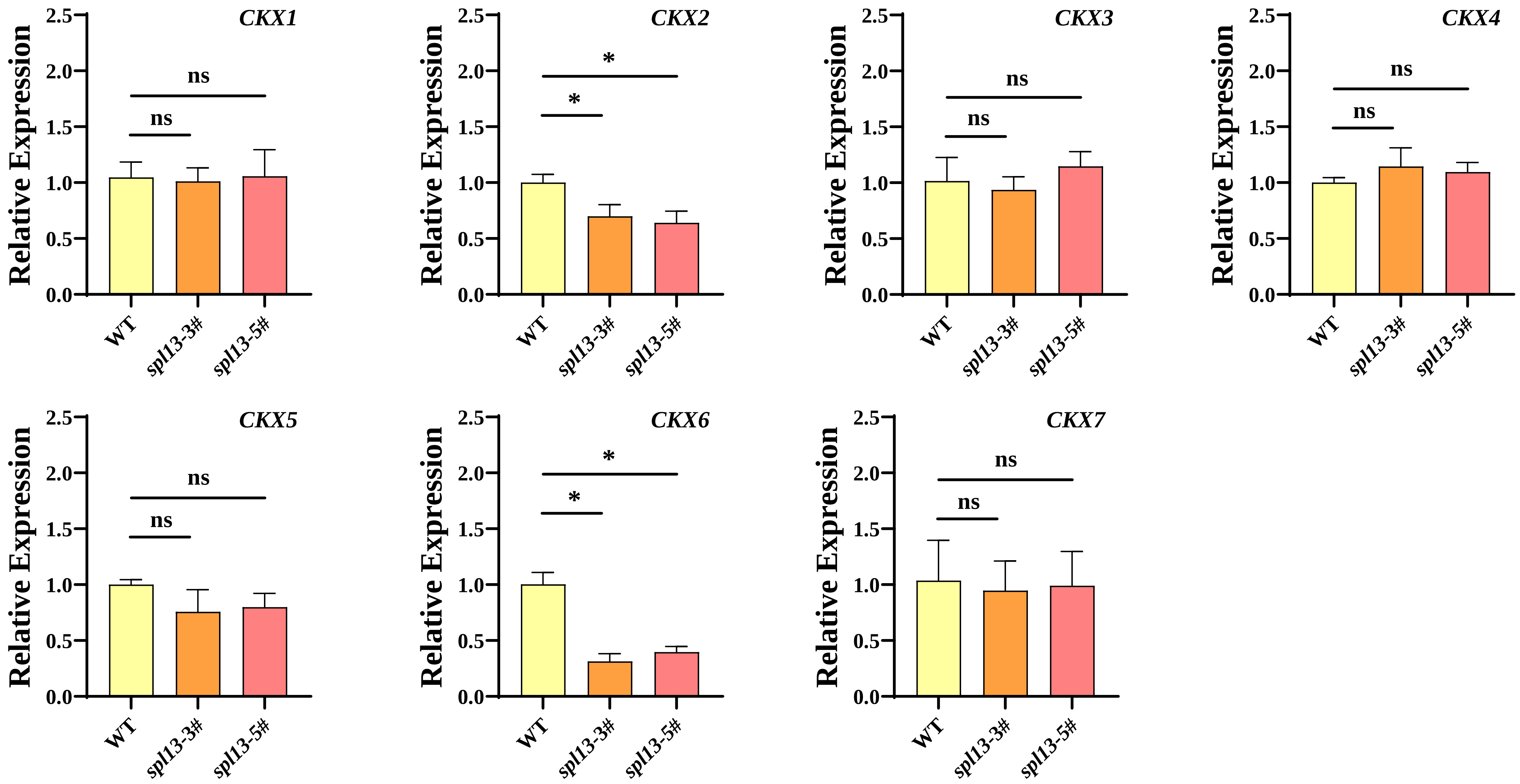

Supplemental Fig. S10. Expression analysis of *CKX1-CKX7* in shoots of WT and *spl13* mutants. Plants in the 6-leaf stage were used in the experiment. Values are means of three biological replicates ± SD. The ns indicate insignificant difference according to Student's test ($p < 0.05$). The asterisks indicate significant difference according to Student's test ($p < 0.05$).

Supplemental Fig. S11

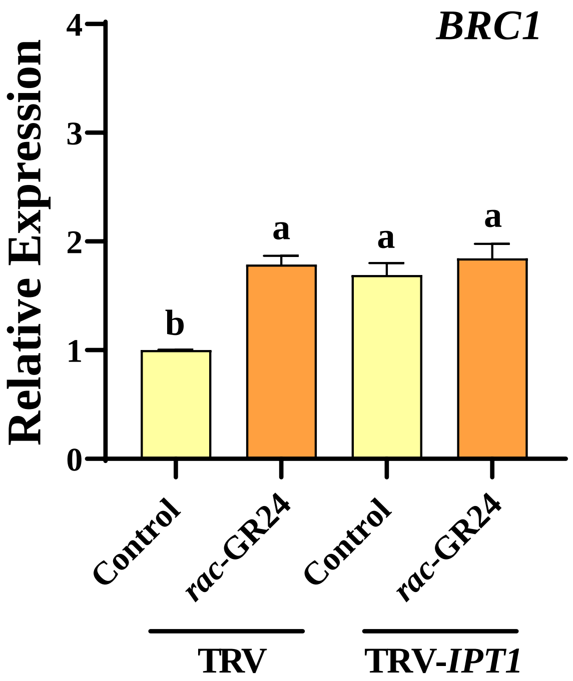

Supplemental Fig. S11. qPCR analysis of relative transcript of *BRC1* in lateral buds. Plants in the 6-leaf stage were used in the experiment. Values are means of three biological replicates ± SD. The different letters indicate significant difference according to Tukey's test ($p < 0.05$).

Supplemental Fig. S12

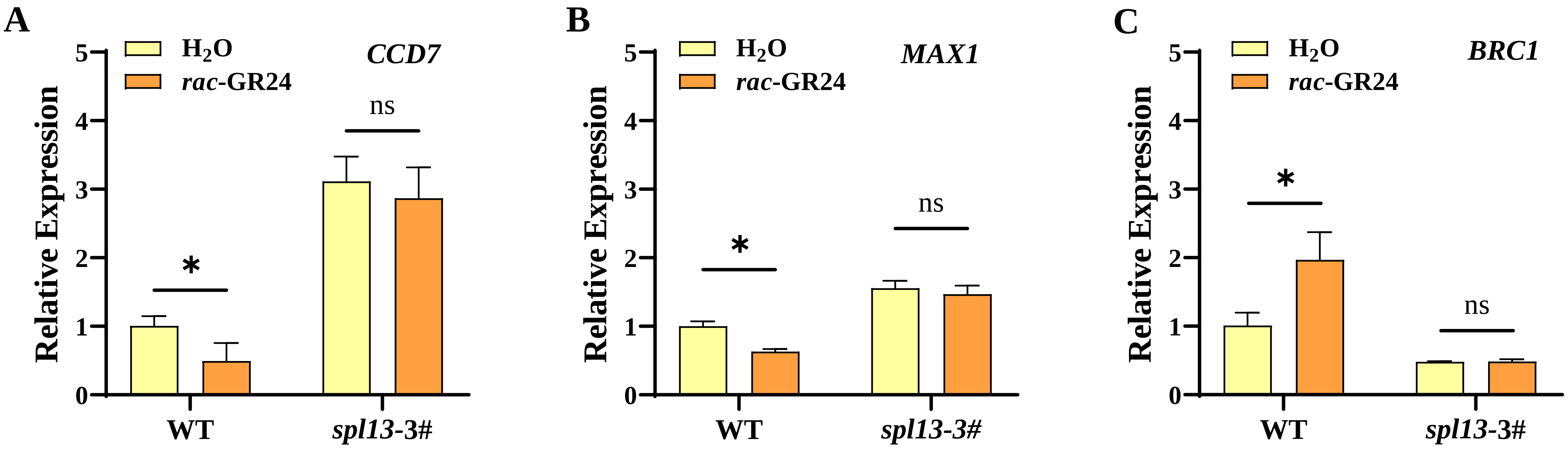

Supplemental Fig. S12. (A and B) Effects of application of *rac*-GR24 on the expression of *CCD7* and *MAX1* in shoots of WT and *spl13* mutant. (C) Effects of application of *rac*-GR24 on the expression of *BRC1* in lateral buds of WT and *spl13* mutant. Plants in the 6-leaf stage were used in the experiment. Values are means of three biological replicates ± SD. The ns indicate insignificant difference according to Student's test ($p < 0.05$). The asterisks indicate significant difference according to Student's test ($p < 0.05$).

Supplemental Fig. S13

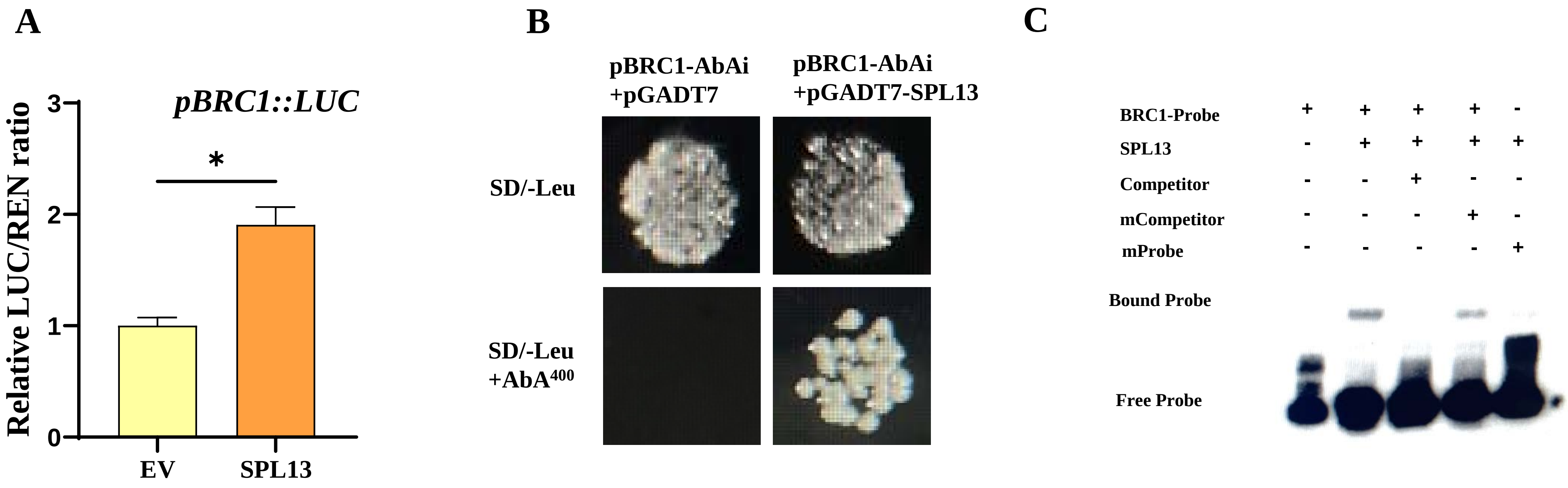

Supplemental Fig. S13. SPL13 promotes the transcription of *BRC1*. (A) Dual-luciferase assay for the regulation of *BRC1* expression by SPL13. (B) Y1H analysis of SPL13 binding to the P_{BRC1}. (C) EMSA analysis of SPL13 binding to the *BRC1*. Values are means of six biological replicates ± SD. The asterisks indicate significant difference according to Student's test ($p < 0.05$).

Supplemental Fig. S14

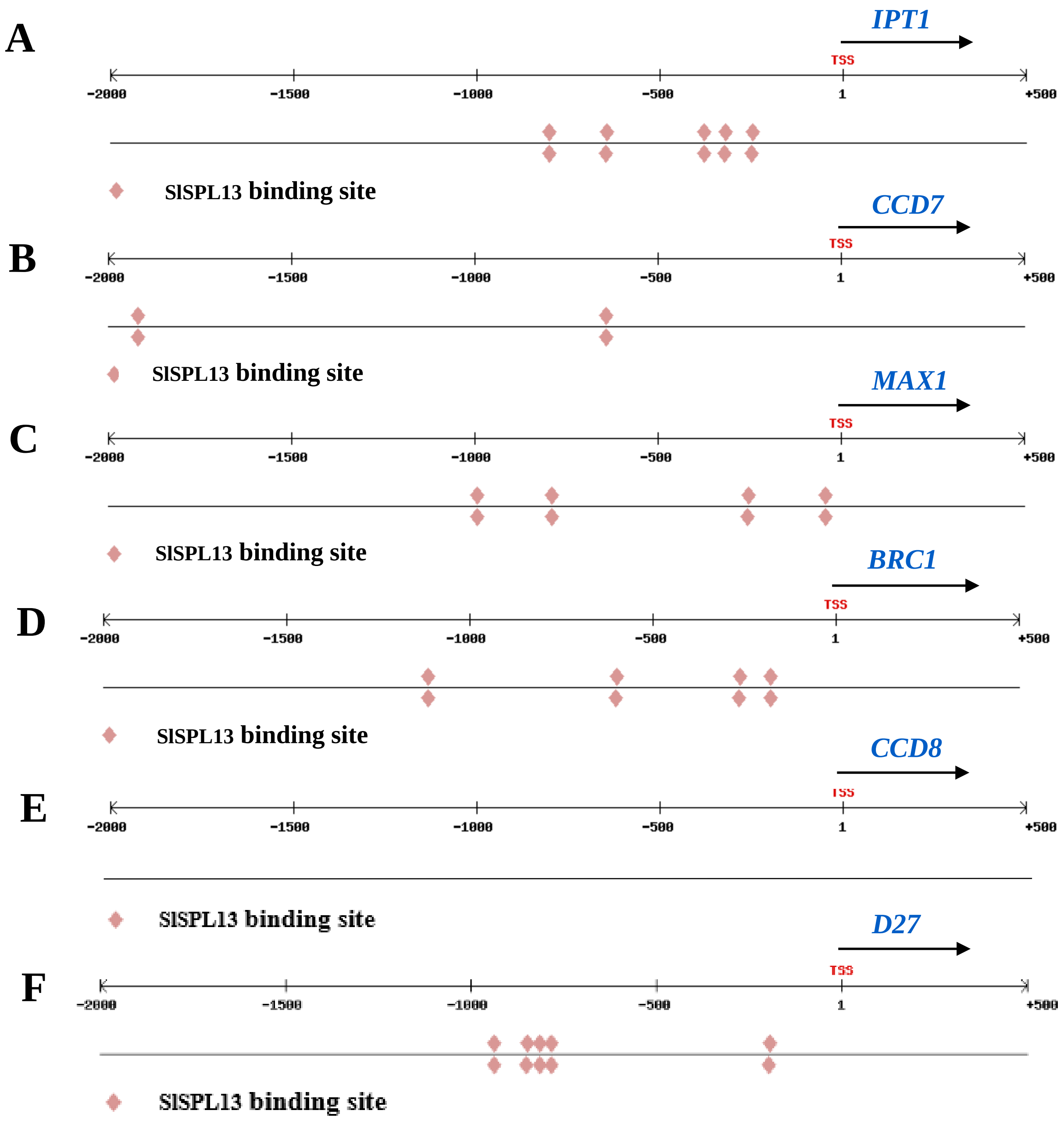

Supplemental Fig. S14. Identification of SPL binding motif in the promoters of *IPT1* (A), *CCD7* (B), *MAX1* (C), *BRC1* (D), *CCD8* (E) and *D27* (F).

Supplemental Fig. S15

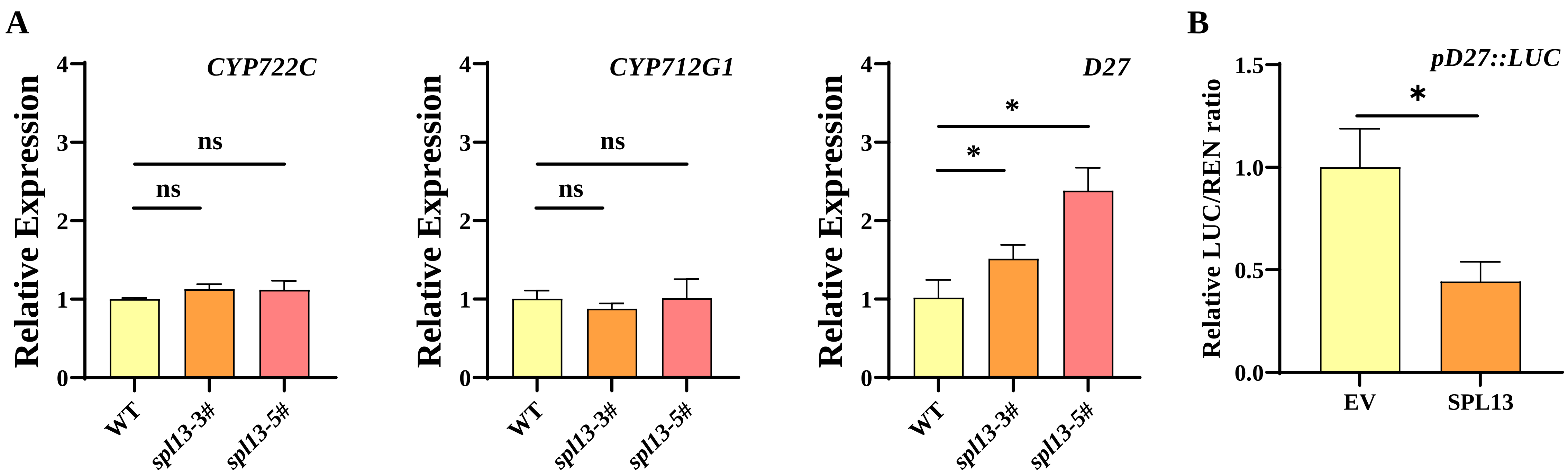

Supplemental Fig. S15. (A) Expression analysis of *CYP722C*, *CYP712G1* and *D27* in roots of WT and *spl13* mutants. (B) Dual-luciferase assay for the regulatory effect of SPL13 on the expression of *CCD7*. Plants in the 6-leaf stage were used in the experiment. Values are means of three biological replicates ± SD. The ns indicate insignificant difference according to Student's test ($p < 0.05$). The asterisks indicate significant difference according to Student's test ($p < 0.05$).

Supplemental Fig. S16

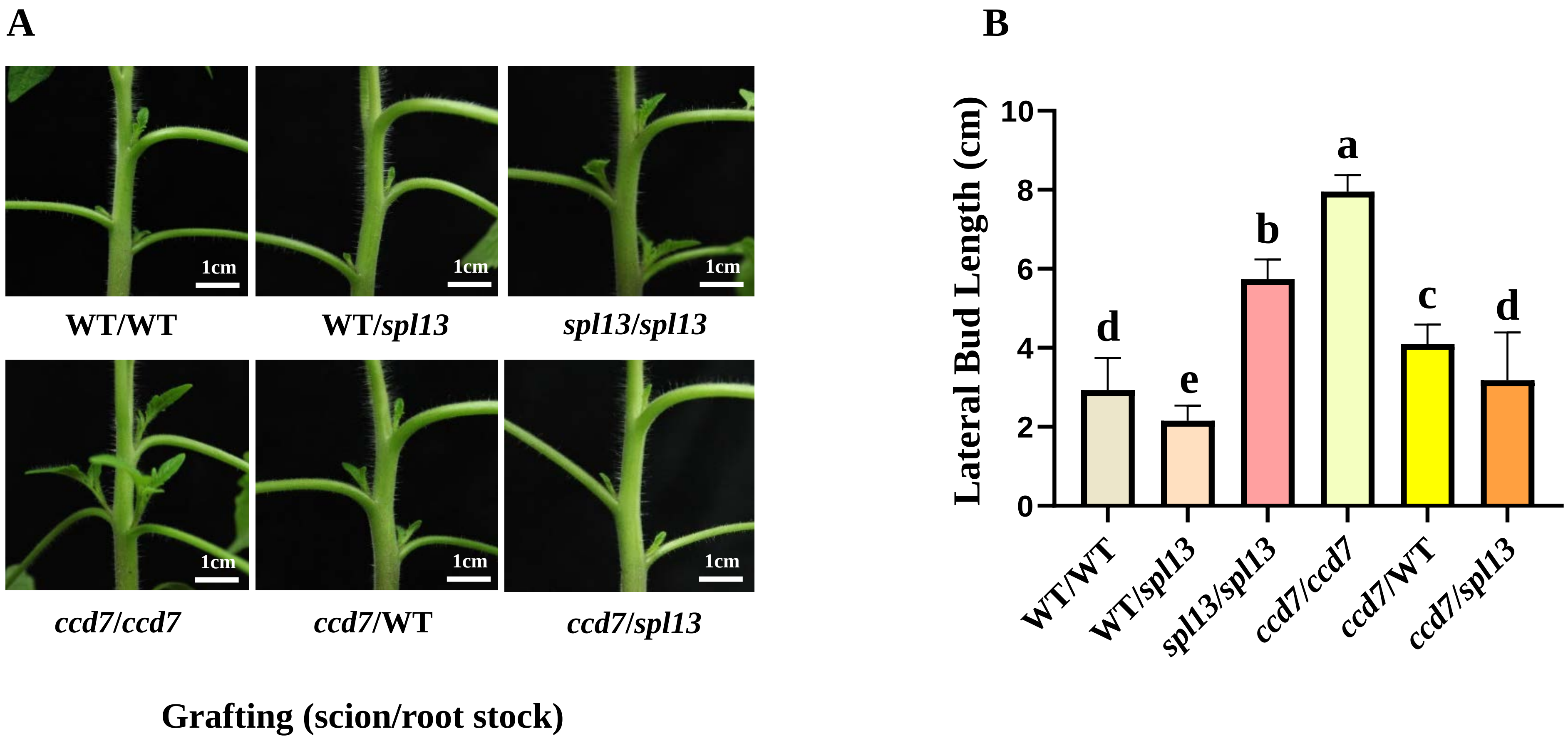

Supplemental Fig. S16. Effects of grafting on the lateral bud outgrowth. (A) Bud outgrowth phenotypes of different grafting combination between WT, *ccd7* and *spl13*. (B) Total lateral bud length. Plants in the 6-leaf stage were used in the experiment. Values are means of four biological replicates ± SD. The different letters indicate significant difference according to Tukey's test ($p < 0.05$).

Supplemental Table

Table S1 Primers used for vector construction

Vector		Primer
**pTRV2-*IPT1***	**Forward**	5’-GTGAGTAAGGTTACCGAATTCTGAGATCGTTTTTTCATATTCATTGTA-3’
	**Reverse**	5’-CGTGAGCTCGGTACCGGATCCTAAATGATGAACAACGCCGCT-3’
**pTRV2-*IPT2***	**Forward**	5’-AGAAGGCCTCCATGGGGATCCGGTTGATGCTATTGCACCTGTATT-3’
	**Reverse**	5’-GAGACGCGTGAGCTCGGTACCTCCACTCTTTGTATGTTCCATCCA-3’
**pTRV2-*IPT4***	**Forward**	5’-AGAAGGCCTCCATGGGGATCCCATCGAGGCATTTGTCCACAA-3’
	**Reverse**	5’-GAGACGCGTGAGCTCGGTACCATCGCTTCCTCTAGCATCCTCTC-3’
**AtU6-sgRNA-AtUBQ-Cas9-*CCD7***	**Forward**	5’-GATTGTCCTCCAAAACTCTTGCCAC-3’
	**Reverse**	5’-AAACGTGGCAAGAGTTTTGGAGGAC-3’
**AtU6-sgRNA-AtUBQ-Cas9-*CCD8***	**Forward**	5’-GATTGCTTCCTGACATGTTTGATCA-3’
	**Reverse**	5’-AAACTGATCAAACATGTCAGGAAGC-3’
**AtU6-sgRNA-AtUBQ-Cas9-*SPL13***	**Forward**	5’-GATTGTCGCCGGCATAAAGTTTGTG-3’
	**Reverse**	5’-AAACCACAAACTTTATGCCGGCGA-3’
**pET-32a-*SPL13***	**Forward**	5’-GCCATGGCTGATATCGGATCCATGGAATCATCATCATCGTCATCA-3’
	**Reverse**	5’-GTGGTGGTGGTGGTGCTCGAGGTCCCACATAAAGGCTAGTGTTTG-3’
**AD-*SPL13***	**Forward**	5’-CGCTCTAGAACTAGTGGATCCATGGAATCATCATCATCGTCATCA-3’
	**Reverse**	5’-GATAAGCTTGATATCGAATTCGTCCCACATAAAGGCTAGTGTTTG-3’
**pAbAi-*IPT1***	**Forward**	5’-CTTGAATTCGAGCTCGGTACCTGCATAATTGTTTGTATTATCTAATATATATGAA-3’
	**Reverse**	5’-ATACAGAGCACATGCCTCGAGTTAACGATAATCGGATATAGTTATTTTTCG-3’
**pAbAi-*CCD7***	**Forward**	5’-CTTGAATTCGAGCTCGGTACCACCGAAAGGAAAGAAAAAAATAAAA-3’
	**Reverse**	5’-ATACAGAGCACATGCCTCGAGCAAACTATCTTATATATATATATATATATAATAATATATGTAAGTTTAC-3’
**pAbAi-*MAX1***	**Forward**	5’-CTTGAATTCGAGCTCGGTACCATACTATATATTAAATTCCCTTAGTTTCTTGTTG-3’
	**Reverse**	5’-ATACAGAGCACATGCCTCGAGGACGTTTGACTATATATATATATATATATATATGTATGTACAT-3’
**pAbAi-*SPL13***	**Forward**	5’-CTTGAATTCGAGCTCGGTACCCAGACTTGTTCCTTTACACTTGTCTCT-3’
	**Reverse**	5’-ATACAGAGCACATGCCTCGAGCTACTGTCGGCTGAAGTCTAATGATT-3’
**pAbAi-*BRC1***	**Forward**	5’-CTTGAATTCGAGCTCGGTACCTCCCTTTTTTTTTTATCTCTCTTTCA-3’
	**Reverse**	5’-ATACAGAGCACATGCCTCGAGTTGTGGAGTAGCTGTAGTTGAGGC-3’
**SK-*SPL13***	**Forward**	5’-CGCTCTAGAACTAGTGGATCCATGGAATCATCATCATCGTCATCA-3’
	**Reverse**	5’-GATAAGCTTGATATCGAATTCGTCCCACATAAAGGCTAGTGTTTG-3’
**LUC-*IPT1***	**Forward**	5’-TCGACGGTATCGATAAGCTTGGCACACATTTTGAACTATTTAATAAATA-3’
	**Reverse**	5’-CGCTCTAGAACTAGTGGATCCATAACAACGATTTTTTTCGTTGTGG-3’
**LUC-*CCD7***	**Forward**	5’-GTCGACGGTATCGATAAGCTTACCGAAAGGAAAGAAAAAAATAAAA-3’
	**Reverse**	5’-CGCTCTAGAACTAGTGGATCCCAAACTATCTTATATATATATATATATATAATAATATATGTAAGTTTAC-3’
**LUC-*MAX1***	**Forward**	5’-GTCGACGGTATCGATAAGCTTTTTGAGGCGGTCTCTAGTAGCG-3’
	**Reverse**	5’-CGCTCTAGAACTAGTGGATCCGACGTTTGACTATATATATATATATATATATATGTATGTACAT-3’
**LUC-*SPL13***	**Forward**	5’-GTCGACGGTATCGATAAGCTTTCAAAATATTTAAATACTTACCAAACATTCA-3’
	**Reverse**	5’-CGCTCTAGAACTAGTGGATCCCAAGACCTTAATCACCTCCTTTTTATG-3’
**LUC-*BRC1***	**Forward**	5’-GTCGACGGTATCGATAAGCTTCACATAATATAACAAATAATTTCAAAATCG-3’
	**Reverse**	5’-CGCTCTAGAACTAGTGGATCCTTGTGGAGTAGCTGTAGTTGAGGC-3’

LUC-*D27*	**Forward**	5'-GTCGACGGTATCGATAAGCTTTAAGTTGGAATAGATGAATATTTAGTAATTAAAA-3'
	**Reverse**	5'-CGCTCTAGAACTAGTGGATCCAGGGGAGAAGTAATTAACTAAGTAGTGCA-3'

Table S2 Primers used for qPCR analysis

	Accession number	Forward primer (5'-3')	Reverse primer (5'-3')
BRC1	Solyc06g069240	TTGATGCTGCTCGCAAGTTT	CAACCTGTGCATCATTGCCT
IPT1	Solyc05g009410	GAGAGAGTGGGTGGGACCTA	TGTCTTTCCCAAATTTCCGT
IPT2	Solyc04g007240	AGCAGCCATGGAGATAAAGG	CGTTTCTGTTGCATCCACTC
IPT3	Solyc01g080150	ACGTGCAATTGGAGTACCAG	GCAAGCCAATTTGCATGTAT
IPT4	Solyc09g064910	GATGCATCGATGAACGTGCT	GGGACACCAATGGCTTTACG
IPT5	Solyc11g066960	CAGTACAGAAATTGGCAGCAA	TCTATGACCACGACCCTGTC
IPT6	Solyc12g014190	GGCTTCTTGATGTGGGTCTT	TCCTCCATCTTCCCTACACC
LOG1	Solyc11g069570	TATGCACCAGAGGAAAGCAG	CAATAGCCCTACCGGTTTGT
LOG2	Solyc09g007830	ACCGAGCTGGTTGAGAAACT	GAACTTGAGGGTAGCCAAGC
LOG3	Solyc04g081290	GGCTATGGGACATTGGAAGA	TGACAAAGCCTTCATCAACAG
LOG4	Solyc01g005680	ATGATGGTGGATGCAATGTT	AAAGCATCAGCTCACTAGCCA
LOG5	Solyc08g062820	AACCATGGAGGAGACTCTGG	CCCTGGCTTAATGAAACCTT
LOG6	Solyc10g084150	CACCATCGTCAAAGGAGCTA	GTGCCTGAGGGTAACCAAGT
LOG7	Solyc10g082020	CCCTGGTGGCTATGGTACTC	AAGCCTTCATCAACAGCCTT
LOG8	Solyc06g075090	CCTGGTGGCTATGGAACTTT	CTTCCTCCACTGCTTTGTCA
CYP735A1	Solyc02g094860	AGCAGTTCGTATGGGAATGA	AGTGGTTTCATGTCCAGCAA
CYP735A2	Solyc02g085880	TGGAATGTACGAGTGGGATG	TATTTCAGCGGTGAGTCGAG
CKX1	Solyc08g061930	CCTATCCTCTCCTGCGTAGC	TTTGTGCCACCATTTGATTT
CKX2	Solyc01g088160	TGCAAATCCTTCATCTAGCG	CCTCCATTGTTCCTGTGTTG
CKX3	Solyc08g061920	TGACGATCCCTCCACTGTAA	CCACTACCTCTAGCCGCTTC
CKX4	Solyc04g080820	TGAGGTCAGCATTGGAGAGT	GGTCCCTCCATTCTCTTTGA
CKX5	Solyc04g016430	CCTCATCCATGGCTAAACCT	ATAGGCCCATGGCTAGTGTC
CKX6	Solyc12g008900	AAAGGAATGTGGGATGTTCC	ATGGGTCCATTTGTCTTGTTT
CKX7	Solyc10g079870	CAGGAAGAGTGGAAGCATCAT	AATCCTCTGTCCTGGAGCAA
CCD7	Solyc01g090660	ATGGATCTTCAATTTGTATC	ACGACCCGAAGTGAAACG
CCD8	Solyc08g066650	CCTTCAACGGCAAGGATGTC	AAGGCCTCTTAGCACCACAA
MAX1	Solyc08g062950	GGTGTTACATTGTCTCGCCC	CTCCAAGAGCCAACCAAACC
CYP722C	Solyc02g084930	GATGCGATGTGCGATATGCT	CACGTGCCTTGATGCCTTTA
CYP712G1	Solyc10g018150	ATGAATGGAGAGACGCGGAT	TCCACCACCAAATGGGACAT
D27	Solyc09g065750	AAAGGGCATTCCCTAAGCCT	TCACAAGGTCCAACTAGCCA
SPL1	Solyc02g077920	TTTGGCAGGTCACAATGAGG	GAGAGCATGCTTAACCCACA
SPL2	Solyc04g045560	ATTGGAAGCAGCCTTTCAGC	AATTCACCATCTCGGCGTTT
SPL3	Solyc10g009080	TCCAGGAGAAGGGTCAAGTT	TGACAGAAGAGAGAGAGCGT
SPL4	Solyc07g053810	CTGTCAACAATGCAGCCGAT	TTCTTCGCCTTTCGTTGTGT
SPL5	Solyc07g062980	GGGAAGCCAGGCCACATA	TCCAGAGATGTCAGCAGTGT
SPL6a	Solyc03g114850	GCGTTGAACTGCTGTAGTGT	TGAGACAGCACCATGAGGAA

SPL6b	Solyc05g012040	GGTGGGCCTACCATGGATTT	CATTGCGGATGTTGACCCTT
SPL6c	Solyc12g038520	CTCCTAGTGCTCACCACCTT	GTGTTCCAATTGACCCTTTGC
SPL7	Solyc01g080670	GGTCAGAATGCACTGCTGTT	GCTCCCTTAGTAACCATGCC
SPL8a	Solyc10g018780	CAGTGCAGCAGGTTCCATTT	CTGATGAACAATGAGCTCCTGA
SPL8b	Solyc01g090730	GCAGATCACAACCGGAGAAG	TGCTGGTATGTTCTTTGCCT
SPL10	Solyc05g015510	AATTCAGCGAGGCTTTCGTC	GAGTCTTGAGTGCTCTCCCA
SPL12a	Solyc01g068100	TGTACAGGCCTGCAATGTTC	CCCTGAAGAGGAGAGCAACA
SPL12b	Solyc05g053240	ACTGGCCACTGGGATTGTAG	GCAGGCAATTAGCAGTGCAT
SPL13	Solyc05g015840	TCACATGTCAGCAACAGCAG	ATATCCGCAGAAAGCAAGGC
SPL15	Solyc10g078700	CTCCTGATCTCGGTTTGGGT	AGAGTCCAGTGCACATTCTGA
ACTIN	Solyc11g005330	TGTCCCTATTTACGAGGGTTATGC	CAGTTAAATCACGACCAGCAAGAT
UBI3	Solyc01g056940	TCGTAAGGAGTGCCCTAATGCTGA	CAATCGCCTCCAGCCTTGTTGTAA
